# Identification of Potential Antiviral Hops Compounds against Chikungunya Virus

**DOI:** 10.3390/ijms24043333

**Published:** 2023-02-07

**Authors:** Tsvetelina Mandova, Marielena Vogel Saivish, Leonardo La Serra, Mauricio Lacerda Nogueira, Fernando Batista Da Costa

**Affiliations:** 1 AsterBioChem Research Team, School of Pharmaceutical Sciences of Ribeirão Preto, University of São Paulo, Avenida do Café s/n, Ribeirão Preto 14040-020, SP, Brazil; 2 Gilson Purification, 22 rue Bourseul, ZI du Poteau, 56890 Saint Avé, France; 3 Departamento de Doenças Dermatológicas, Infecciosas e Parasitárias, Faculdade de Medicina de São José do Rio Preto (FAMERP), São José do Rio Preto 15090-000, SP, Brazil; 4 Virology Research Center, Ribeirao Preto Medical School, University of São Paulo—USP, Ribeirão Preto 14049-900, SP, Brazil

**Keywords:** natural products, hops, acylphloroglucinols, virology, arbovirus, chikungunya, countercurrent chromatography

## Abstract

Chikungunya virus (CHIKV) is an arthropod-borne virus that belongs to the genus *Alphavirus* (family Togaviridae). CHIKV causes chikungunya fever, which is mostly characterized by fever, arthralgia and, sometimes, a maculopapular rash. The bioactive constituents of hops (*Humulus lupulus*, Cannabaceae), mainly acylphloroglucinols, known as well as *α*- and *β*-acids, exerted distinct activity against CHIKV, without showing cytotoxicity. For fast and efficient isolation and identification of such bioactive constituents, a silica-free countercurrent separation method was applied. The antiviral activity was determined by plaque reduction test and was visually confirmed by a cell-based immunofluorescence assay. All hops compounds demonstrated a promising post-treatment viral inhibition, except the fraction of acylphloroglucinols, in mixture. *β*-acids fraction of 125 µg/mL expressed the strongest virucidal activity (EC_50_ = 15.21 µg/mL), in a drug-addition experiment on Vero cells. Hypothesis for mechanism of action were proposed for acylphloroglucinols based on their lipophilicity and chemical structure. Therefore, inhibition of some steps of the protein kinase C (PKC) transduction cascades was also discussed.

## 1. Introduction

**Figure 1:**
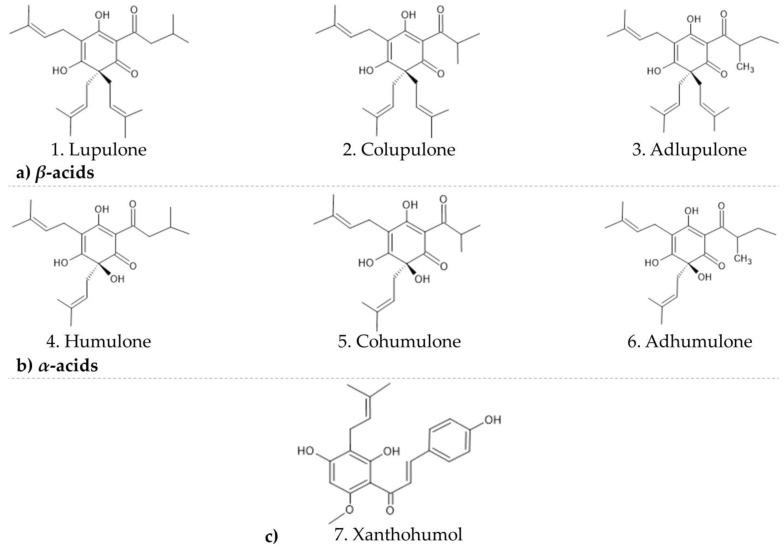
Chemical structures of tested compounds from hops on chikungunya virus strain: (**a**) *β*-acids, (**b**) *α*-acids and (**c**) xanthohumol.

Vector-borne diseases are human illnesses caused by parasites, viruses and bacteria that are transmitted by vectors. Chikungunya virus (CHIKV) is an arthropod-borne virus that causes an infectious disease, transmitted by the same vector as dengue, yellow fever, and Zika [1,2]. CHIKV is a single-stranded positive-sense RNA, an enveloped alphavirus around 11.8 kb long that belongs to the arthritogenic Old World alphaviruses. The genome encodes six structural proteins—C (nucleocapsid), E3, E2, E1, 6K, and its translational frameshift product TF; and four nonstructural proteins—nsP1, nsP2 (helicase), nsP3, and nsP4 (polymerase) [3,4]. The disease is mainly transmitted to humans by the *Aedes aegypti* and *A. albopictus* mosquitoes [5,6]. Most CHIKV cases are characterized by an acute infection with fever, myalgia and arthralgia [3,7]. Despite the fact that CHIKV originated in Africa, the virus is extremely present in South America and Brazil, in particular. Between 2013 and 2019 in Latin America countries, the Pan American Health Organization reported more than six hundred deaths associated with CHIKV infection, with possible strong underestimation of deaths caused by the virus in the region. Meanwhile, the highest number of CHIKV-related deaths in the Americas, 214 cases, was reported in Northeast Brazil, Ceará state [7,8]. Unfortunately, no efficient antivirals against CHIKV infection are available so far. Therefore, it is important to deepen our understanding of molecular mechanisms that regulate CHIKV infections, like virus host cell interactions or replication strategies, to understand viral pathogenesis [9,10,11,12,13]. At the same time, historically, natural products (NP) have played a key role in the drug discovery process, especially for cancer and infectious diseases. In general, NP are characterized by enormous scaffold diversity and structural complexity, showing greater molecular rigidity when compared with synthetic compound libraries, something that can be valuable in drug discovery tackling protein–protein interactions [13,14,15]. In this context, the current study aimed to evaluate the antiviral potential of well-known compounds from the hops crop *Humulus lupulus* L. from the Cannabaceae family, a supercritical inflorescence extract. Commercial hop production occurs predominantly in temperate climates and is an essential ingredient in brewing, adding bitterness and aroma to beer [16]. Beer is often considered to be a “functional beverage” for several reasons, including its prolonged shelf life and absence of microbial contamination through the use of hops, which provide a characteristic mechanism of microbial inhibition [17,18]. Previous phytochemical investigations of hop inflorescences revealed that its characteristic secondary metabolites are acylphloroglucinols (*α*- and *β*-acids) and their derivatives as well as flavones and chalcones, besides volatile oil terpenoids. *β*-acids described in this study are lupulone (1), colupulone (2), and adlupulone, (3) and *α*-acids are humulone (4), cohumulone (5), and adhumulone (6) (Figure 1) [19]. Few searches in the past investigated whether crude hop extracts or purified hop components, representing every major chemical class of hop compound, have antiviral activity. Some of the hop constituents were tested against different kinds of viruses; nevertheless, only iso-*α*-acids and xanthohumol showed low to moderate inhibition. The *β*-acids did not have any activity assigned to any of the tested viruses; therefore, none of them was an alphavirus species [20,21]. In this work, for the first time we show that hops compounds demonstrated a promising virucidal activity against CHIKV. We suggest several hypotheses explaining the antiviral activity. The formation of dimers between *α*- and *β*-acids could explain some obstructions and decrease of activity during the later steps of CHIKV processing. Similarity with the diacylglycerol and phorbol can be found in the structure of acylphloroglucinols and therefore explain the similar activity and potential activation of the protein kinase C pathway, and thus antiviral activity on CHIKV. Some similarities with the structure of proven inhibitors of CHIKV are discussed. Finally, we suggest that the acylphloroglucinols from hops could impact the CHIKV replication process by inhibiting membranes-related processes during viral protein formation, interaction, cleavage, and regrouping.

## 2. Results

In order to achieve an efficient liquid/liquid separation by CCS with high resolution of constituents of the supercritical hops extract, several solvent systems were evaluated on a small scale in test tubes. A solvent system is considered suitable when *K* values of the constituents to be isolated range in the area of 0.5–2 [22,23]. *K* values are expressed as the ratio of the concentration of an analyte in the stationary phase to its concentration in the mobile phase. Finally, after the solvent search, the solvent system consisting of heptane-ethyl acetate-ethanol-NH_4_Ac buffer (1.0 M) at 7:3:5:5 (*v*/*v*) was selected as most convenient and used in the separation process. After CCS was run, five fractions were retained and chosen, keeping some diversity in the ratio of the congeners from *α*- and *β*-phloroglucinols, besides one fraction consisting of cohumulone (major *α*-acid) and one enriched on the major chalcone xanthohumol. All compounds were identified after UHPLC-MS/MS (data not published) analysis and fitted retention time after HPLC analysis [24]. The fraction of cohumulone was analyzed by NMR ^1^H and 2D experiments and the confirmation of structure was made by comparing with literature data [18]. The fractions and respective compounds kept proceeding to the in vitro tests were defined as follows:

- Fraction 3–*β*-acids
- Fraction 9–*α*-and *β*-acids
- Fraction 20–*α*-acids
- Fraction 24–major *α*-acid cohumulone
- Fraction 35–major chalcone xanthohumol and phenolic compounds (flavonoids)

**Figure 2:**
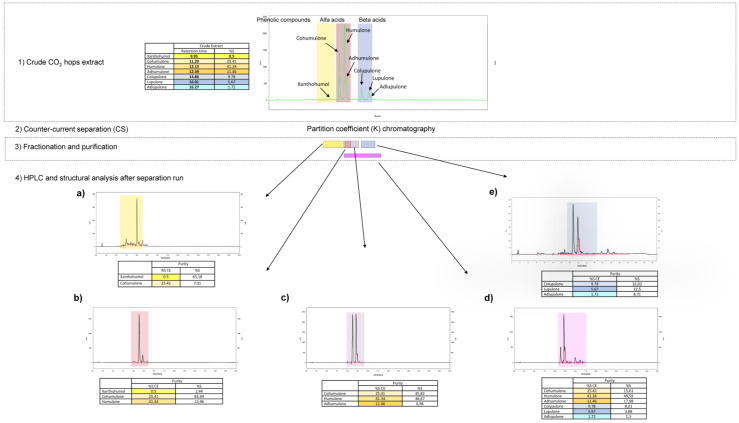
Steps of the purification process of the hops compounds: (1) HPLC chromatogram identifying compounds to purify in *H. lupulus* crude extract, with table indicating retention times of each identified compound and surface area in %; (2) CCS instrument purification step; (3) fractionation and purification of compounds; (4) HPLC chromatograms of each isolated compound, with table indicating their quantities in the crude extract assessed with the purification range: (**a**) xanthohumol and phenolic compounds; (**b**) cohumulone; (**c**) *α*-acids; (**d**) *α*- and *β*-acids; (**e**) *β*-acids.

The applied steps of hops purification and HPLC characterization of enriched fractions with corresponding yield percentage are described in Figure 2. The lack of one hundred percent analytically proved individual pure compounds’ fractions allowed us to observe some synergy effect between congeners. Synergy can occur through a variety of mechanisms, including (i) pharmacodynamic synergism through multi-target effects; (ii) pharmacokinetic synergism through modulation of drug transport, permeation, and bioavailability; (iii) elimination of adverse effects; and (iv) targeting disease resistance mechanisms [25].

### 2.1. Assessment of Host Cell Toxicity

To evaluate the cytotoxic effect of hop compounds, we set the maximum concentration for toxicity assessment to 250 µg/mL by compound. The Vero cell toxicity was analyzed 72 h post-treatment using a MTT cytotoxicity test assay, which is a colorimetric assay that measures the metabolic activity of living cells. All five compounds displayed cell viability above 50% at the highest concentration of 250 µg/mL; thus, no CC_50_ value could be measured, and no selectivity index (SI) could be presented (Table 1).

### 2.2. Hops Compounds Inhibit Plaque-Forming Unit of Chikungunya

**Figure 3:**
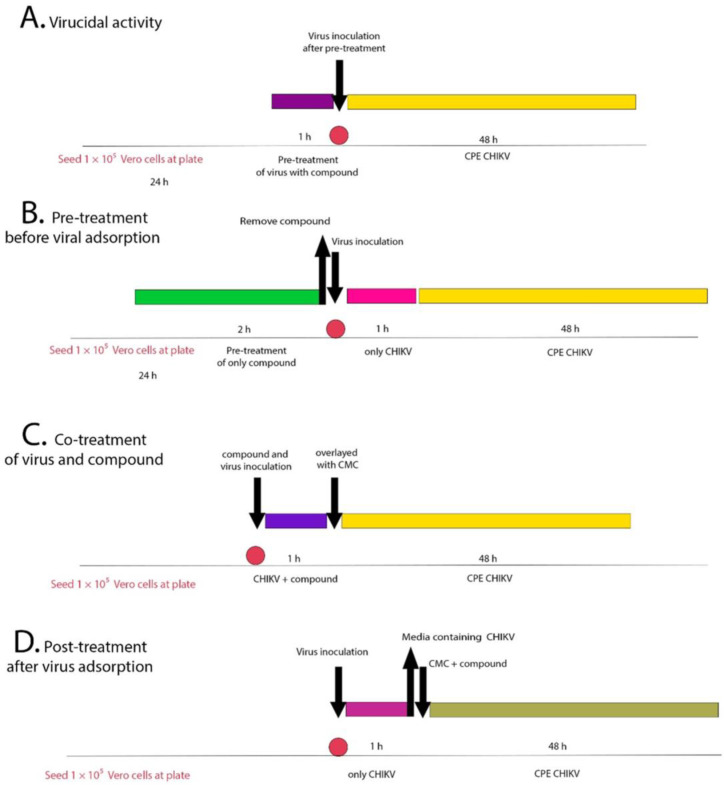
Schematic of antiviral assay at different stages of virus infection. (**A**) Virucidal assay that consists in pre-treatment of virus with compounds followed by inoculation of the treated virus to the cells; (**B**) pre-treatment assay: addition of the compounds at 2 h prior to virus infection; (**C**) co-treatment assay: virus incubated with the compounds at 37 °C; (**D**) post-treatment assay: addition of the compounds at 1 h post-viral infection; CPE—cytopathic effect, CMC—carboxymethyl cellulose (the colors were used to differentiate each step of inoculation, there are no specific color meanings).

**Figure 4:**
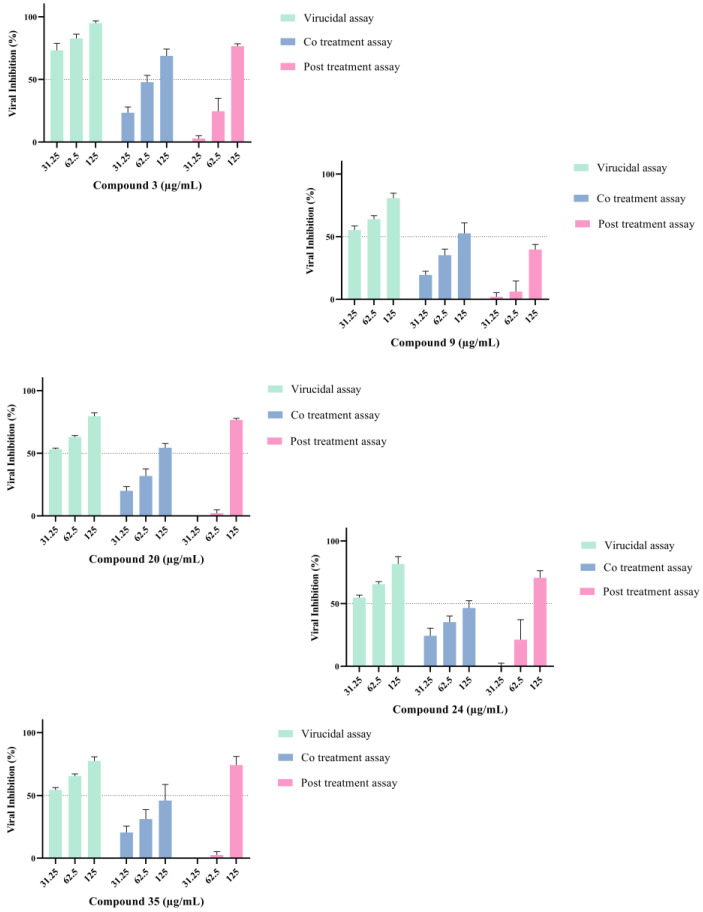
Percentage of viral inhibition under different experimental conditions. The inhibitory effect of the five compounds on CHIKV replication was assessed in a dose-dependent manner, with a constant challenge of 50 PFU per well. Data represented % of virus inhibition compared to untreated control as mean ± SD (*n* = 3), each time by triplicate.

The antiviral activity of all five fractions—acylphloroglucinols—in mixture or alone, and the chalcone fraction were evaluated by performing a plaque-forming unit inhibition assay to determine the effective concentration of 50% (EC_50_) (Table 2). All results in detail are provided in annexed supplementary file data. The formation of plaques corresponds to a full infection cycle, from entry to egress of newly formed viral particles. To explore which step(s) of the viral life cycle were blocked by the compounds, time-of-drug-addition experiments were performed. The pre-treatment examines whether the substance could block the viral receptor to inhibit viral attachment to the host cells or if it could induce production of antiviral host factors. This assay did not show any promising result. Therefore, they are not shown and not discussed further. The inhibitory effect of the compound on CHIKV replication was assessed in a dose-dependent manner (Figure 3), with a constant challenge of 50 PFU per well. Vero cells were infected with CHIKV and simultaneously treated with hops fractions at concentrations ranging from 31.25 to 125 µg/mL in two-fold serial dilutions, and viral replication was assessed 48 h post-infection hpi. We conducted a plaque formation assay in a dose-dependent manner by using each of the five tested compounds to observe plaque-forming inhibition against the virus. After the virus-induced cytopathic effect of 2 days, the cells were fixed, and plaques were visualized by crystal violet staining and then quantified (Table 2). All five fractions showed reduction of plaque formation at post-treatment and co-treatment assays, mainly in highest concentration tested (Figure 4).

### 2.3. Hops Compounds Strongly Affect Post-Entry Steps of CHIKV Infection

**Figure 5:**
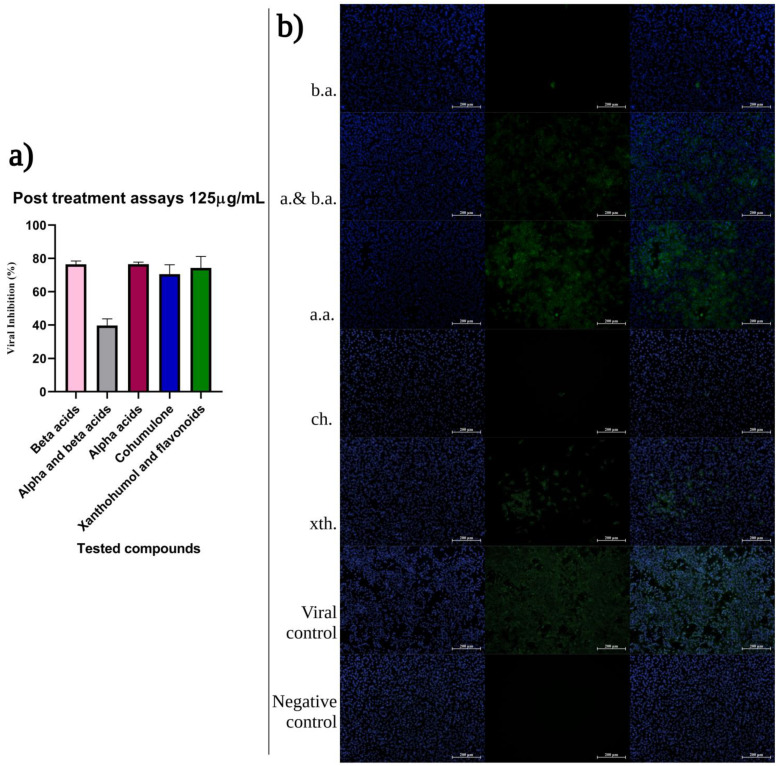
(**a**) Majority of hops compounds protect cells against CHIKV infection. Vero cells were treated independently with 125 µg/mL at MOI 0.1 for 1 h, in agitation. The virus-containing medium was removed and replaced with the compound solubilized in 1% carboxymethyl cellulose (CMC). Infected cells were incubated for 48 hpi. (**b**) Immunofluorescent images of CHIKV-infected Vero cells under post-treatment condition with the compound of interest at 125 μM. The cells were challenged with MOI = 0.1 of CHIKV. Negative control corresponds to cells not infected by the virus, and CHIKV-infected cells without compound were used as positive control. The CHIKV was detected by IFA, with CHIKV MIAF antibody stained with 488 ALEXA antibody and then stained with DAPI to visualize nuclei (blue). Images are representative of three independent experiments. Scale bars are 200 µm. (*abb*. b.a.—*β*-acids; a and b.a—*α*-and *β*-acids; a.a.—*α*-acids; ch.—cohumulone; xth.—xanthohumol and flavonoids). Visual sensitivity was increased to shallow contrast by Adobe Photoshop 2021.

Treatment of virus-infected cells during the entire post-inoculation period examines the antiviral effect of compounds during the latest steps, such as genome translation and replication, virion assembly, and virion release from the cells. It was found that, if added after virus infection, hops compounds cause relatively strong reduction of CHIKV replication (*p* < 0.0001), around 80%, except the fractions where the *α*-and *β*-acids are in mixture, where the inhibition decreased twice (Figure 5).

### 2.4. Hops Compounds Moderately Affect Early Stages of CHIKV Infection

**Figure 6:**
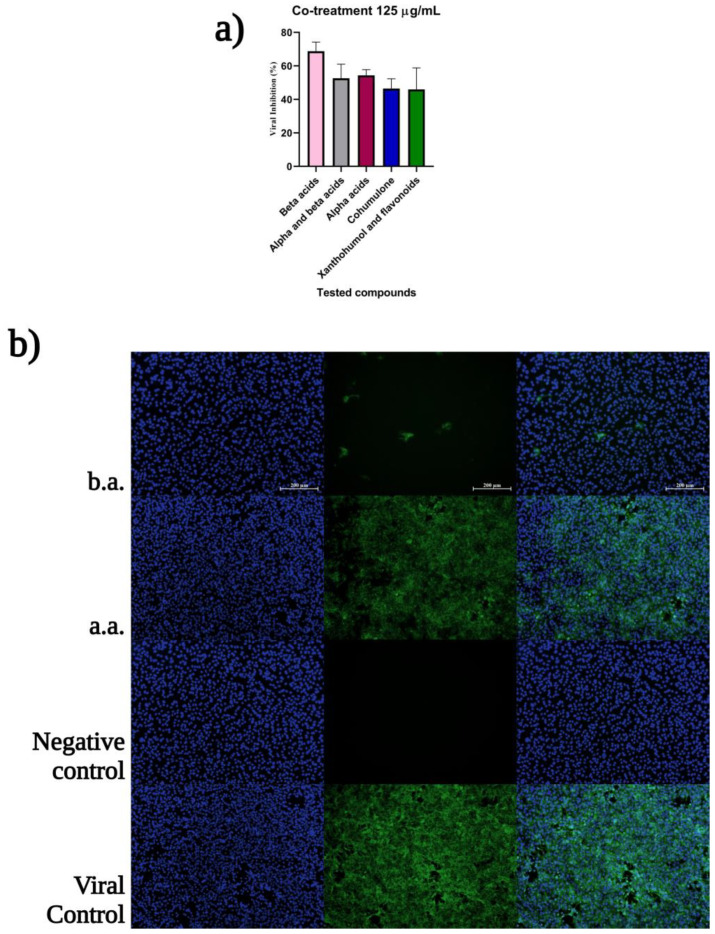
(**a**) *β*-acids has virucidal activity and blocks CHIKV entry to the host cells during the co-treatment assay; (**b**) The immunofluorescence assay in 125 µg/mL at MOI 0.1, shows significant difference between inhibition of the entry stages by *β*-acids (b.a.) and *α*-acids (a.a.) where the growth lag is closer to the viral control. Images are representative of three independent experiments. Scale bars are 200 µm. Visual sensitivity was increased to shallow contrast by Adobe Photoshop 2021.

Co-treatment of cells and virus during virus inoculation examines the compounds on the virus entry steps, including virucidal (neutralizing) activity and blockade of viral attachment and penetration to the cells. The neutralizing activity of hops compounds described by virucidal activity was significant for all compounds, at the highest concentration of 125 µg/mL. The *β*-acids expressed the strongest activity during the virucidal (close to 100%) and co-entry steps of viral inoculation, around 70% (Figure 6).

## 3. Discussion

**Figure 7:**
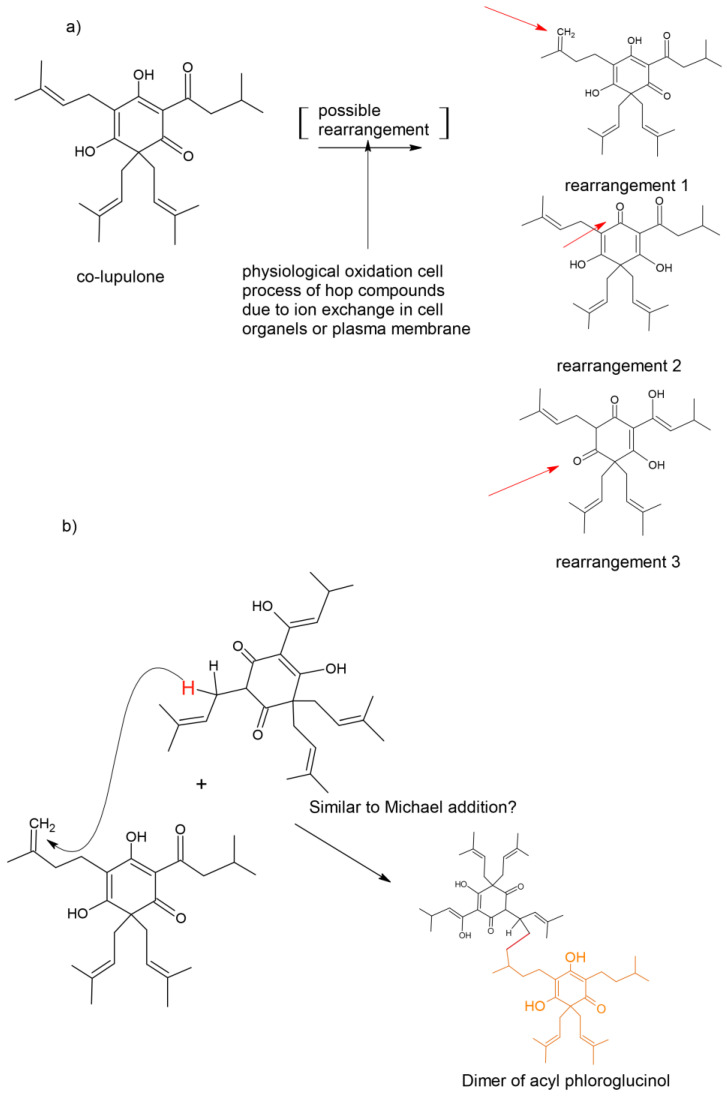
(**a**) Possible oxidation process once the compounds are exposed internally in the cells. Red arrows indicate where changes occurred; (**b**) Plausible interactions of acylphloroglucinols to form dimers, inspired after the proposal of biosynthetic pathways in (23) observed in *L. scoparium*.

The current study was the first to assess the effects of hops compounds against chikungunya virus via different methods of CHIKV inoculation on Vero cells. The identification of new antiviral strategies relies on a better understanding of CHIKV host–cell interactions and on the elucidation of the molecular mechanisms and cellular pathways co-opted by the virus to become a successful human pathogen. It is well known that time-of-addition assay can provide a preliminary understanding of the infectious phase upon which compounds act. On the other side, hops are known primarily as raw materials, supplying characteristic bitterness and aroma to beer. Hydrophobic compounds from hop cones are responsible for bitterness (*α*- and *β*-acids), besides the characteristic hoppy aroma of beer, due to the volatile oil. In addition, hop cones contain several biologically active phenolic compounds and other constituents that have attracted the attention of researchers. The hydrophobic acylphloroglucinols showed strong effect on post-inoculation steps after CHIKV, except when the two congeners groups are in mixture, when the activity decreases twice. Acylphloroglucinols are characterized by a phloroglucinol core substituted by an acyl side chain, such as isovaleryl, methylbutyryl, acetyl, benzoyl, and phenylpropanoyl. Meanwhile, phloroglucinols could couple with terpenes, triketones, or other phloroglucinols to build more complex natural products, which exhibit multiple biological activities. Structures with unprecedented skeletons, such as phenylpropanoyl–phloroglucinol dimers, were isolated from *Leptospermum scoparium* (Myrtaceae), well known as the source of manuka honey, an evergreen shrub distributed widely in New Zealand [26]. By having similar structure, we are making the hypothesis that dimers could be formed after interaction between *β*- and *α*-acids from hops. This plausible interaction is shown in Figure 7, inspired by the biosynthetic pathways suggested for the dimeric acylphloroglucinol derivatives from *L. scoparium* [26]. The formation of dimers between *α*- and *β*-acids could explain some obstructions and decrease of activity during the later steps of CHIKV processing. More studies need to be carried out to understand if the different nature of *α*- and *β*-acids intensifies dimer formation.

**Figure 8:**
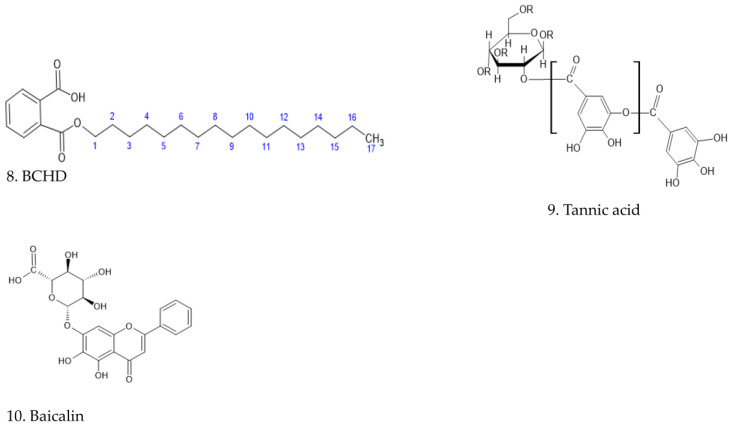
Structure of benzene-1-carboxylic acid-2- hexadeconate (BCHD (8)), tannic acid (9), and baicalin (10).

Scuotto et al. [27] identified several indole derivatives of umifenovir (arbidol) having a 10-fold improved anti-CHIKV inhibitory activity. The presence of an electron-withdrawing substituent, such as a carbonyl group of the phenol ring, yields molecules with more pronounced antiviral effect. A study by Sangeetha et al. [28] exploring the anti-CHIKV capacity of extracted compounds from leaves of *Tectona grandis* Lin. (Verbenaceae) showed that compounds like benzene-1-carboxylic acid-2-hexadeconate (BCHD) (8) (Figure 8) were 76.46 times more active than ribavirin to treat RSV (Respiratory Syncytial Virus) infection, hepatitis C, and some viral hemorrhagic fevers [28]. The possible interaction of lipophilic chain compounds, like in the case of hops *β*-acids, could be either by inhibiting the replication or by preventing the maturation of CHIKV virus (post-entry stage of inoculation). One of the possible hypotheses could be the interaction of the alkyl chain reinforcing the lipophilic nature and therefore the interaction with some of the non-structural proteins (nsp1 & nsp 4). The nsp1 is a membrane-associated protein that bears methyltransferase and guanyltransferase activities and is involved in the capping of (+) RNA genome and the attachment of the viral replication complex to cytoplasmic membranes. Nsp4 protein contains the RNA-dependent RNA polymerase involved in genome replication and transcription.

Meanwhile, another study underlined that the presence of hydroxyl groups could be crucial for the anti-CHIKV activity of new antivirals. Tannic acid (TA) (9) (Figure 8) is a weak acid (pKa = 10) that may also affect the culture cells, in that it has an inactivation showing a close relation to phenolic hydroxyl groups, since the displacement of the hydroxyl groups by methoxy makes the chemicals ineffective and reduction of these groups makes their effects weaker [4,9].

Further phenolics, hydroxylated natural products structures such as flavonoids, are capable of interacting with structural and non-structural proteins of chikungunya. Ten residues in the active site of nsP3 were identified as the major interaction sites for various flavonoids. Finally, one π-π stacking interaction between baicalin (10) (Figure 8) with nsP3 residue Tyr114 was also observed. Four others—Leu108, Tyr142, Ser110, and Thr111—were proved to interact as well, at some points. Considering the involvement of CHIKV nsP3 in the intracellular replication cycle, the results suggest that baicalin could potentially interfere with the post-entry stage(s) of CHIKV infection [10,29]. All those compounds described in the literature have similarities with acyl phloroglucinols hops compounds and xanthohumol. Therefore, our results could support the previous data of structural preferences like lipophilic chains or hydroxyl groups, suggesting that the predominant activity of hops compounds is due to their effect, probably by acting on the virus at later stages [4].

**Figure 9:**
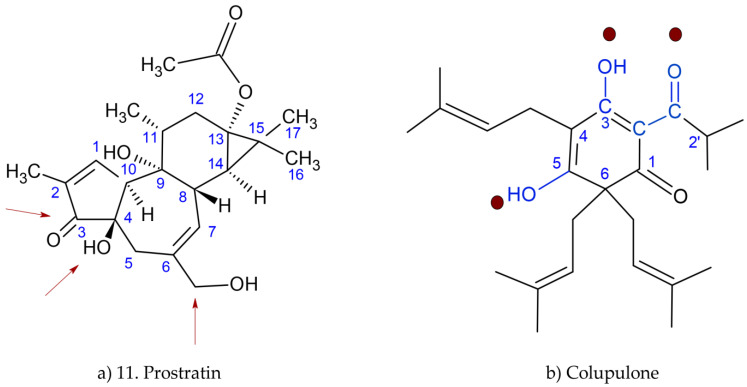
Structure of (**a**) prostratin (11) and (**b**) colupulone indicating groups of plausible interaction with PKC (red dots).

Another plausible suggestion explaining the antiviral activity on CHIKV of acylphloroglucinols is their close analogy with some inhibitors of protein kinase C. Prostratin (a short ester of 12-deoxyphorbol) [30,31] is a phorbol esters originally isolated from the oil fraction of the Asian purging croton (*Croton tiglium* L.) [32,33] and the tropical plant *Homalanthus nutans* (G.Forst.) Guill*,* a native species from the Pacific islands [34], both from the spurge family (Euphorbiaceae). Prostratin is a potent activator of protein kinases C (PKCs), enzymes that play important roles in several signal transduction cascades. The activity of PKCs is diacylglycerol- and calcium-ions-dependent [35,36]. Studies proved that the interaction of phorbols with PKC depends on their radicals and requires an optimal combination of hydrogen bonds and hydrophobic interactions. A cysteine-rich region would be the target site for phorbol. The compound binding happens by replacing a water molecule, therefore establishing hydrogen bonds through the oxygen atoms attached to carbons C-3, C-4, and C-20 (Figure 9a), indicated as red arrows. The importance of hydrophobic acyl chains of phorbol esters lies in the formation of a complex with the protein kinase enzyme and thus attaching to the membrane [37].

The pharmacophoric model for prostratin requires a lipophilic region, three groups capable of forming hydrogen bonds, and adequate distance and orientation between the lipophilic region of the ester chains and the ring systems of the phorbol backbone. The similarity with the acylphloroglucinol could eventually be the reason for the activity of hops compounds on the Vero cells and expressing inhibition in the chikungunya virus replication process (Figure 9b) [37]. The red dots within the colupulone structure suggest where the plausible interaction between acylphloroglucinol and PKC could occur. Reference to the structure of the phorobol derivative and diacylglycerol (DAG) was used.

Kramer et al. [36] explained that the phorbol derivatives activity lies with the similarity with the diacylglycerol. This similarity and activation of PKC pathway explain the irritant activity of these compounds. The skin contact of phorbol ester causes inflammation, as witnessed by swelling, warmness, redness, and pain. These symptoms are the consequences of its stimulation of histamine release, activation of integrins, stimulation of lymphocytes (IL-2-mediated expansion), and the release of proteases, cytokines, and reactive oxygen and nitrogen species by neutrophils and macrophages. Similarity with the DAG and phorbol can be found in the structure of acylphloroglucinols, and this hypothesis could explain the similar activity and potential activation of PKC pathway, thus antiviral activity on CHIKV. The *β*-acids, by being lipophilic compounds, show affinity for the plasma membrane. This is the mechanism of action for their antibacterial properties. The mechanism of inhibition of beer spoilage bacteria is known. Gram-positive bacterial species can spoil beer, increase turbidity, and produce unpleasant aromatic compounds such as diacetyl or hydrogen sulfide [38]. The mechanism of inhibition of sensitive cells by hop resins was first explained by Simpson et al. [39]. Hops *β*-acids, *α*-acids, and also iso-*α*-acids are incorporated into cell membranes. They act as mobile carrier ionophores, catalyzing processes including the electroneutral influx of undissociated molecules, exchange of protons for divalent cations such as Mn^2+^, and efflux of the resulting complex. Thus, the acyl phloroglucinols from hops could impact the CHIKV replication process by inhibiting the membranes-related process during viral protein formation, interaction, cleavage, and regrouping. This hypothesis is in agreement that the more lipophilic *β*-acids could interact more easily with cellular membranes, including plasma membranes and internal membranes, and therefore inhibit viral cycle.

## 4. Materials and Methods

**Plant material.** The supercritical carbon dioxide extract of *Humulus lupulus* L. was obtained from the company Lychnoflora, Ribeirão Preto, Brazil, with the material duly registered under ATH 01-18. Details on the plant material and origin could be revealed by Lychnoflora company. The extract was stored following the recommendations of the manufacturer—under 20 °C, without freezing. All experiments were performed in accordance with relevant named guidelines and regulations available in the federal universities, the Ministry of Environment, and IBAMA.

**Compounds purification.** Counter-current chromatography was carried out using high-speed counter-current chromatography (HSCCC) Quatro CCCTM MK 5 and MK 6 (AECS-QuikPrep Ltd., London, UK) equipped with two coils holding four PTFE pipe columns. The experiments were made in coil 1, with a theoretical volume of 235 mL, i.d. = 3.2 mm, and a constant-flow gradient HPLC pump. Separations by HSCCC were conducted in the tail-to-head mode (normal phase elution mode) at 30 °C adjusted temperature, 850 rpm coil rotation speed, and flow rate of 4 mL/min; 4 mL fractions were collected. The selected solvent mixture was thoroughly equilibrated in separating funnel at room temperature before proceeding to column charge and the two phases separated shortly before use. Therefore, the multiplayer coil column was first entirely filled with the upper stationary phase. Then, the lower mobile phase was pumped into the inlet of the column at the flow rate of 4 mL/min, while the apparatus was rotated at 800 rpm. After that, the mobile phase was eluted from the head outlet and the two phases had established the hydrodynamic equilibrium throughout the column. Once stabilized, the sample solution was injected through the injection valve. The effluent from the outlet of the column was continuously monitored by TLC analysis and the collected fractions were further analyzed by HPLC. The retention volume Sf = 79%.

**Solvent system search.** During the search of biphasic system, solvents were prepared by mixing, after individual measuring in a graduated cylinder, in suitable proportions (*v*/*v*); all solvents were by degrees added into 15 mL tubes, adding a small quantity of crude extract sample and shaking them vigorously. Once settled until the phases became limpid, an equal amount, around 10 µL, was placed on TLC plate and migrated in a chamber previously saturated with the system heptane-acetone-ethyl acetate 5:1.5:1.5 (*v*/*v*). Further, the TLC analysis–by short wavelength UV light held close to the plate at 255 and 366 nm (for aromatics + conjugated systems), covering the majority of the natural compounds that could be detected in UV. Finally, the revealing was finalized with vanillin solution followed by heating until formation of colored spots.

**HPLC analysis**. Analyses were performed using analytical reverse-phase column (ODSC18 column 20 mm × 250 mm, Shimadzu, Japan); the equipment was a Prominence Shimadzu chromatograph linked to a CBM 20 controller, UV/visible detector SPD-20, LC 6 AD pumps, and automatic fraction collector FCR-10. A mobile phase composed of 0.1% HCOOH in (A) H_2_O and (B) was ACN. The gradient elution was modified as follows: 0–10 min from 35% to 75% B, 10–30 min from 75% to 100% B, which was held for 5 min. The post-running time was 10 min. The flow rate was set at 0.5 mL/min. The column temperature was set at 30 °C. The sample injection volume was 10 µL. The UV acquisitions were carried out in the range 190–500 nm and chromatograms were acquired at 280 nm (for bitter acids) and 270 nm (for prenylchalcones).

**NMR.** The cohumulone fraction was analyzed by NMR to confirm its chemical structure. The ^1^H NMR and ^13^C NMR spectra were acquired on a Bruker Avance III 400 MHz spectrometer for ^1^H and 2D experiments and Bruker Avance 300 MHz spectrometer for ^13^C, using Bruker pulse programs. NMR Fourier transform, integration and peak picking were done with Bruker TopSpin software with spectra referenced to solvent signals as follows: CDCl_3_ (δ 7.26 and 77.0 ppm) and CD_3_OD (δ 3.35, 4.78, and 49.3 ppm). MestReNova Lite CDE software was used to process of the NMR spectra (Supplementary Information).

**Cell culture.** Vero cells were grown in Minimal Essential Medium (MEM) supplemented with 10% heat-inactivated fetal bovine serum (FBS), 100 U.mL-1 of penicillin, 0.1 mg·mL^−1^ of streptomycin, and 0.5 µg·mL^−1^ of fungizone (Gibco, Waltham, MA, USA) and cultured at 37 °C under a 5% CO_2_ atmosphere. C6/36 cells were maintained in Leibovitz-15 medium (L-15) with 10% FBS at 28°C. Chikungunya virus stocks were cultured in C6/36 cells and titrated on Vero cells using plaque-forming assay, described below. Working solutions of compounds were prepared in MEM at the indicated concentrations at the time of use.

**Viral propagation and titration of the stock.** The CHIKV strain is a human isolate (genotype ECSA–strain BHI3762, accession number H804917), and stocks were propagated in C6/36 cells. To determine the viral titer of the stock obtained, a titration was performed in Vero cells. Briefly, Vero cells grown in a 24-well culture plate were infected by 0.1 mL of ten-fold dilutions of supernatants. Following an incubation of 1 h at 37 °C, 0.5 mL of culture medium supplemented with 2% FBS and 1,5% carboxymethylcellulose sodium salt (Sigma-Aldrich, Saint-Quentin-Fallavier, France) were added, and the incubation was extended for two days at 37 °C. The cells were fixed (formaldehyde 10%) and stained with 2% crystal violet diluted in 20% ethanol after removing the media. Plaques were counted and expressed as plaque-forming units per milliliter (PFU·mL^−1^). The value of the viral titer obtained from the viral stock was used to perform all subsequent tests, being properly converted to perform the plate reduction tests to evaluate the antiviral activity.

**Cytotoxicity analysis and determination of the effective concentration (EC_50_)**. Briefly, 4 × 10^4^ Vero cells grown in 96-well plates in MEM were treated with each of the five fractions after a CCC purification. Concentrations ranging from 125 µg/mL to 31.25 µg/mL for 72 h were tested for each of the five fractions. Then, 1 mg/mL of 3-(4,5-dimethyl-2-thiazolyl)-2,5-diphenyltetrazolium bromide (MTT, Sigma-Aldrich, Saint Louis, MI, USA) was added to the cells, and they were incubated for 1 h. Formazan crystals were dissolved in DMSO, and absorbance was determined at 550 nm using a Spectramax Plus Microplate reader (Molecular Devices, Sunnyvale, CA, USA). Results are shown as the percentage of viable cells relative to untreated control cells. All assays were performed three times independently in triplicate. The effective concentration of 50% inhibition (EC_50_) was calculated from a dose-response curve in GraphPad Prism (version 8.00) using four-parameter curve-fitting.

**Definition of Viral Infection Assay Terms.** To explore which step(s) of the CHIKV replication cycle are blocked by fractions, four different treatment conditions were utilized in time-of-drug experiments. First, in a virucidal assay, each fraction was added to the virus as a pre-treatment 1 h prior to inoculation of the treated virus into the cells in order to determine its virucidal or neutralizing activity. Next, a pre-treatment assay involved treating the cells with fractions 2 h prior to viral inoculation. The third assay utilized co-treatment, with fractions and virus simultaneously added to the cells to determine the compound’s effect on virus entry, including virucidal (neutralizing) activity and blockade of viral attachment to and penetration into the cells. Finally, in a post-treatment assay, virus-infected cells were treated during the entire post-inoculation period to determine the antiviral effect of fractions during post-entry steps, such as genome translation and replication, virion assembly, and virion release from the cells. Viral infection experiments were performed in Vero cells seeded in 24-well plates treated with or without fractions.

**Plaque reduction assay.** The plaque reduction assay was performed using a modified protocol previously described. Briefly, Vero cells were seeded in 24-well plates at a density of approximately 1 × 10^5^ and incubated at 37 °C in a humidified atmosphere containing 5% CO^2^ for 24 h. For the virucidal assay, a constant challenge of 50 PFU was treated with each fraction (125–31.25 µM) in final volumes of 200 µL for 1 h at 37 °C. Then, these volumes were added to the confluent cell monolayers; plates were re-incubated for 1 h. Subsequently, the viral inoculum was removed, and a semi-solid overlay was added. For the co-treatment assay, a constant challenge of 50 PFU was treated with fractions (125–31.25 µM) in final volumes of 200 µL and immediately added to the confluent cell monolayers; plates were re-incubated for 1 h. Subsequently, the viral inoculum was removed, and a semi-solid overlay was added. For the post-treatment assay, 200 µL of the viral inoculum (50 PFU) were added to the confluent cell monolayers; plates were re-incubated for 1 h. Subsequently, the viral inoculum was removed and a semi-solid overlay containing fractions in decreasing concentrations (125–31.25 µM) was added. In the pre-treatment assay, cells were treated for 2 h with each of the isolated compounds, one at a time, prior to the CHIKV infection, extensively washed with PBS, and added of CHIKV for 1 h. Then, cells were covered with semi-solid overlay and incubated. For the virucidal pre-, co-, or post-treatment assays, control samples consisting of virus-infected untreated and uninfected cells were also included in each assay. Plates were maintained for 48 h in humidified atmosphere containing 5% CO^2^ with daily observation. After this period, the overlay was removed and cell monolayers were stained with a crystal violet solution, where non-stained lesions (plaques) were quantified and compared to the viral control. The percentage of viral inhibition was calculated by IP = [1- (number of test plates / number of control plates)] × 100.

**Immunofluorescence assay.** Vero cells were plated onto 24-well plates (1 × 10^5^ per well) and incubated at 37 °C with 5% CO2 for 24 h. Cells were infected at a MOI of 0,1 of CHIKV, washed and incubated with compounds for 42 h. Cells were washed and fixed in 4% paraformaldehyde, washed again and permeabilized with 0,05% Triton X-100 (JT Baker). Cells were blocked in 5% BSA (Sigma-Aldrich, St. Louis, MO, USA) and incubated with CHIKV MIAF antibody. AlexaFluor 555 (Abcam, Boston, MA USA) and DAPI were used to stain viral proteins and cell nucleus, respectively. Negative control corresponds to cell not infected by the virus and CHIKV-infected cells without compound were used as positive control.

**Statistical analysis.** Individual experiments were performed in triplicate and all assays were performed three times to confirm the reproducibility of the results. All values were expressed as mean ± SD. Comparison between different concentrations was done by a two-way ANOVA test followed by Tukey’s post hoc test. All statistical tests were done using the software Graph-Pad Prism (version 8.0; GraphPad software, La Jola, CA, USA). *p* values *p* < 0.0001 were considered to be statistically significant.

## 5. Conclusions

In conclusion, we have demonstrated that acylphloroglucinols have significant antiviral activities against CHIKV in vitro, on Vero cells. Viral inoculation, stage-dependent inhibition of hop bitter compounds of chikungunya virus after time-of-drug-addition assay showed that all compounds in the highest concentration express virucidal potential. The most polar acylphloroglucinol bitter compounds demonstrated promising inhibition in post treatment assay. Based on the lipophilicity and chemical structure, the suggestion on protein kinases C (PKC) interactions was proposed. Furthermore, in this work, for the first time we show that hops compounds demonstrate a promising antiviral activity against CHIKV.

## Figures and Tables

**Table 1:** Cell toxicity of hops compounds in Vero cell lineages.

Cell Viability (%) *					
	*β*-acids	*α*-and *β*-acids	*α*-acids	Cohumulone	Xanthohumol and Flavonoids
250 µg	97.6 ± 15.8	95.3 ± 12.1	90.0 ± 11.9	90.9 ± 11.4	96.4 ± 12.1
125 µg	97.9 ± 11.2	96.5 ± 8.8	95.3 ± 8.2	90.1 ± 10.1	97.4 ± 10.7
62.5 µg	107.1 ± 8.0	97.5 ± 7.8	97.2 ± 11.8	79.6 ± 25.9	102.6 ± 15.1
31.25 μg	98.5 ± 14.9	95.7 ± 7.4	89.5 ± 11.0	98.4 ± 17.8	106.6 ± 11.62

* Values with confidence intervals (CI) 95%.

**Table 2:** Inhibition efficacy (EC_50_) of hops compounds in Vero cell lineages.

	Inhibition Efficacy (µg/mL)				
	EC_50_				
	*β*-acids	*α*-and β-acids	*α*-acids	Cohumulone	Xanthohumol and Flavonoids
Virucidal	15.21	19.74	20.81	20.74	19.12
Co-treatment	41.87	41.23	44.35	30.69	36.72
Post-treatment	68.60	72.29	68.07	64.16	70.78

## Data Availability

The authors confirm that the data supporting the findings of this study are available within the article and its Supplementary Materials.

## Supplementary Materials

The following supporting information can be downloaded at: https://www.mdpi.com/article/10.3390/ijms24043333/s1.
