# Supplementary material for: Identification of Potential Antiviral Hops Compounds against Chikungunya Virus
Source: Int J Mol Sci. 2023 Feb 7;24(4):3333. doi: 10.3390/ijms24043333 (PMC9966109; doi:10.3390/ijms24043333)
Supplement: Supplementary file 1 [file ijms-24-03333-s001.zip › ijms-2168767-supplementary.pdf]

# Supplementary Information

# Identification of Potential Antiviral Hops Compounds against Chikungunya Virus

**Tsvetelina Mandova [1,2,*], Marielena Vogel Saivish [3,*], Leonardo La Serra [4], Mauricio Lacerda Nogueira [3]
and Fernando Batista Da Costa [1]**

[1] AsterBioChem Research Team, School of Pharmaceutical Sciences of Ribeirão Preto, University
of São Paulo, Avenida do Café s/n, Ribeirão Preto 14040-020, SP, Brazil

[2] Gilson Purification, 22 rue Bourseul, ZI du Poteau, Saint Avé 56890, France

[3] Departamento de Doenças Dermatológicas, Infecciosas e Parasitárias, Faculdade de Medicina de São José
do Rio Preto (FAMERP), São José do Rio Preto 15090-000, SP, Brazil

[4] Virology Research Center, Ribeirao Preto Medical School, University of São Paulo—USP,
Ribeirão Preto 14049-900, SP, Brazil

* Correspondence: tmandova@gilson.com (T.M.); marielenasaivish@gmail.com (M.V.S.)

**Table of content**

Purification part ... 3

Solvent system search ... 3

CCC parameters – rotation and flow rate ... 4

HPLC profiles (purity described as %S) and physicochemical data of the isolated compounds ... 14

Antiviral part ... 20

MTT assay ... 20

Plaque reduction test results ... 22

Virucidal activity ... 22

Co treatment ... 24

Post treatment ... 26

Pre treatment ... 28

Immunofluorescence – Virucidal activity ... 31

**List of tables**

Table S1: Solvent systems in their ratio (v/v) tested for extrapolation on CCC ... 4

Table S2: Study of relationship between rotation, flow rate and pressure of CCC equipment, for experimental conditions optimization ... 5

Table S3: Summary table of the CCC first experimental run conditions ... 6

Table S4: Summary table of the CCC second experimental run conditions ... 6

Table S5 : Quantitative analyses of the selected fractions for further investigation ... 12

Table S6: Selected fraction after HPLC analysis and their annotation in the manuscript ... 13

Table S7: HPLC retention times and purity (%S) of all analysed compounds in the crude $CO_2$ extract ... 14

Table S8: HPLC purity (%S) of xanthohumol and cohumulone in the crude $CO_2$ extract and in fraction 35, respectively ... 14

Table S9: HPLC purity (%S) of xanthohumol, cohumulone and humulone in the crude $CO_2$ extract and in fraction 24, respectively ... 14

Table S10: 1H (400 MHz) and 13C (300 MHz) NMR data of cohumulone in chloroform D ... 16

Table S11 : HPLC purity (%S) of cohumulone, humulone and adhumulone in the crude $CO_2$ extract and in fraction 20, respectively ... 19

Table S12 : HPLC purity (%S) of all described and studied alpha and beta acids, in the crude $CO_2$ extract and in fraction 9, respectively ... 19

Table S13: HPLC purity (%S) of colupulone, lupulone and adlupulone, in the crude $CO_2$ extract and in fraction 3, respectively ... 19

**List of figures**

Figure S1: HPLC profile of the crude $CO_2$ extract with annotation by chemical formula for each peak ........ 7
Figure S2: TLC profiles of CCC first run, from fraction 1 to 27 (separated into two parts), in 366 nm, 254 nm and after TLC visualization reagent, respectively; 5µL application for each fraction, cohumulone is encircled ........ 10
Figure S3: HPLC profile of fraction 24; 84 % S for cohumulone (25.41% S crude extract) ....... 10
Figure S4: TLC profiles of CCC second run, from fraction 1 to 35 (separated into two parts), after TLC visualization reagent, 254 nm and 366 nm respectively ; 5µL application for each fraction. All fractions of interest used in the study are encircled ........ 12
Figure S5: Schematic representation of the crude $CO_2$ hops extract; red lines indicate each of the fractions selected for further investigation ........ 12
Figure S6 : Chemical structure of xanthohumol (7) ........ 14
Figure S7: Chemical structure of cohumulone (5) ........ 15
Figure S8 : $^{1}$H NMR spectrum of cohumulone (5) (400 MHz, chloroform D) ........ 17
Figure S9: $^{13}$C NMR COSYgp spectrum of cohumulone (5) (300 MHz, chloroform D) ........ 17
Figure S10: $^{13}$C NMR HSQC spectrum of cohumulone (5) (300 MHz, chloroform D) ........ 18
Figure S11: $^{13}$C NMR HMBC spectrum of cohumulone (5) (300 MHz, chloroform D) ........ 18
Figure S12: Chemical structure of alpha acids ........ 19
Figure S13: Chemical structure of beta acids ........ 20

# Purification part

## Solvent system search

A range of systems with various proportions of solvents was developed to ease this choice; 23 variations of the heptane/ethyl acetate/methanol/water biphasic liquid system were labeled A to Z. This range proved to be extremely useful and became the popular Arizona (AZ) liquid system. However, users often replace the heptane with hexane and methanol with ethanol.

| | Hexane | AcOEt | EtOH | W | comments | Adducts | | | |
|---|---|---|---|---|---|---|---|---|---|
| 1 | 2 | 2 | 2.5 | 3 | affinity to upper phase | Cu(Ac)2 40mmol solution | Beta cyclodextrin 40 mmol | FeCl3 40mmol | CuSO4 40mmol |
| 2 | 1 | 3 | 3 | 3 | Emulsion | 0.50 μl | 0.50 μl | 0.25 μl | 0.50 μl |
| | | AcOEt | MeOH | W | | | | | |
| 6 | | 2 | 4 | 5 | weak emulsion | 0.50 μl | 0.50 μl | 0.25 μl | 0.50 μl |
| | | | | | | | | | |
| 7 | | ACN | Hexane | W | | | | | |
| | | 2 | 4 | 2.5 | | 0.50 μl | 0.50 μl | 0.25 μl | 0.50 μl |
| 8 | Hexane | AcOEt | EtOH | W | | | | | |
| | 1.5 | 2.5 | 2.75 | 2.5 | | 0.50 μl | 0.50 μl | 0.25 μl | 0.50 μl |

*Table S1: Solvent systems in their ratio (v/v) tested for extrapolation on CCC*

Finally, a buffer was used to insist on pKa difference of the α- vs β- acids with the system Heptane/ Ethyl Acetate/ Ethanol/ Buffer NH4Ac (1.0 M) in 7/3/5/5 (v/v).

After, solvent search and established conditions the solvent system of Heptane/ Ethyl Acetate/ Ethanol/ Buffer NH4Ac (1.0 M) in 7/3/5/5 (v/v) was prepared. The solvent systems were equilibrated using a separatory funnel. Following solvent/buffer equilibration and separation the pH of the buffer was adjusted accordingly (pH 5). The CCC was completely filled with stationary phase. The CCC was plumbed to elute in a tail-to-head direction, therefore the lower phase was stationary (ascending mode).

**CCC parameters – rotation and flow rate**

In the case of hydrodynamic instrument, the rotation in general is between 600 and 900 rpm (for analytical tool; max 150 psi (around 10 bar)).

Relations between rotor spinning speed, flow rate and pressure

| Rotation in rpm | Flow rate (mL /min) | Mode | Pressure (psi) |
|---|---|---|---|
| 200 | 10 | ASC | 90 |
| 700 | 10 | ASC | 90 |
| 700 | 15 | ASC | 140 |
| 600 | 1.8 | ASC | 100 |
| 710 | 1.5 | ASC | 100 |
| 650 | 1.5 | ASC | 100 |
| 700 | 4.0 | ASC | 120 |

*Table S2: Study of relationship between rotation, flow rate and pressure of CCC equipment, for experimental conditions optimization*

Too high pressure was observed in descending mode due to the mobile phase being aqueous phase, therefore having too high density and thus not appropriate for the elution. Finally, ascending mode was retained as optimal.

Thus, conditions for 700 rpm and 3.5 mL/min, 100 -120 psi were applied

| 1st RUN CCC<br>**Solvent system:** 7/3/5/5 Heptane/ AcOEt/EtOH / W(NH4Ac 1.0M) | |
|---|---|
| Parameter | Value |
| Mode | Ascending |
| Flow rate | 4 mL/min |
| Injected amount (g) | 1 g |
| Injected in volume (mL) | 10 mL (5mL mobile phase & 5mL stationary phase) |
| Rotation | 700 |
| Pressure | 150 psi |
| Temperature | 29 °C |
| Death volume (equilibrium) | 49 mL |
| Volume before the compounds get out of the column | 50 mL |
| Volume fraction | 3 mL , 25,26,27 – 15 mL |

| | |
|---|---|
| Fractions bleeding total | 5 mL |

*Table S3: Summary table of the CCC first experimental run conditions*

Searching to ameliorate the separation, after TLC evaluation a second run was scheduled, this time decreasing the flow rate by 0.5 mL having at the end 3.5 mL.

| **2nd RUN CCC**<br>**Solvent system:** 7/3/5/5 Heptane/ AcOEt/EtOH / W(NH4Ac 1.0M) | |
|---|---|
| parameter | Value |
| Mode | Ascending |
| Flow rate | 3.5 mL/min |
| Injected amount (g) | 1 g |
| Injected in volume (mL) | 10 mL (5mL mobile phase & 5mL stationary phase) |
| Rotation | 700 |
| Pressure | 150 psi |
| Temperature | 29 °C |
| Death volume (equilibrium) | 35 mL |
| Volume before the compounds get out of the column | 50 mL |
| Volume fraction | 3 mL, 25,26,27 – 15 mL |
| Fractions bleeding total | 5 mL |

*Table S4: Summary table of the CCC second experimental run conditions*

The TLC analysis **-** by short wavelength UV light held close to the plate at 255 nm and 366 nm (For aromatics + conjugated systems), covering the majority of the natural compounds that could be detected in UV light. Finally, the revealing was finalized with vanillin solution purposing the revelation was of aldehyde, ketones and alcohols.

HPLC analysis

| fraction | quantity | comments |
|---|---|---|
| 24 | 8.4 mg | NMR analysis |

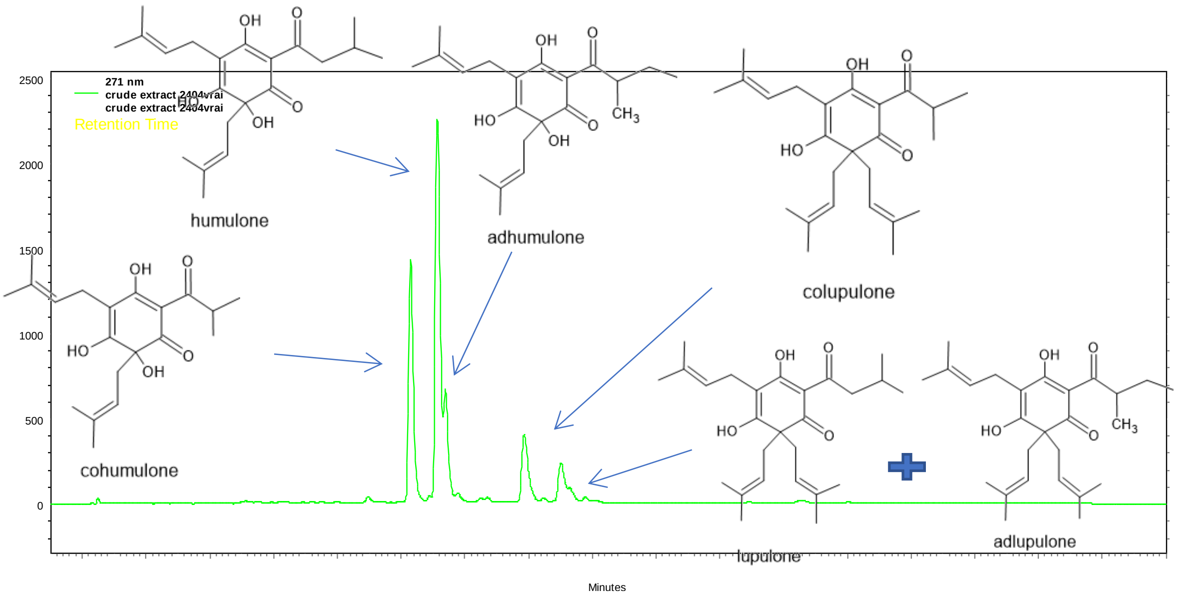


*Figure S1: HPLC profile of the crude $CO_2$ extract with annotation by chemical formula for each peak*

**FIRST RUN CCC – TLC & HPLC**

366 nm

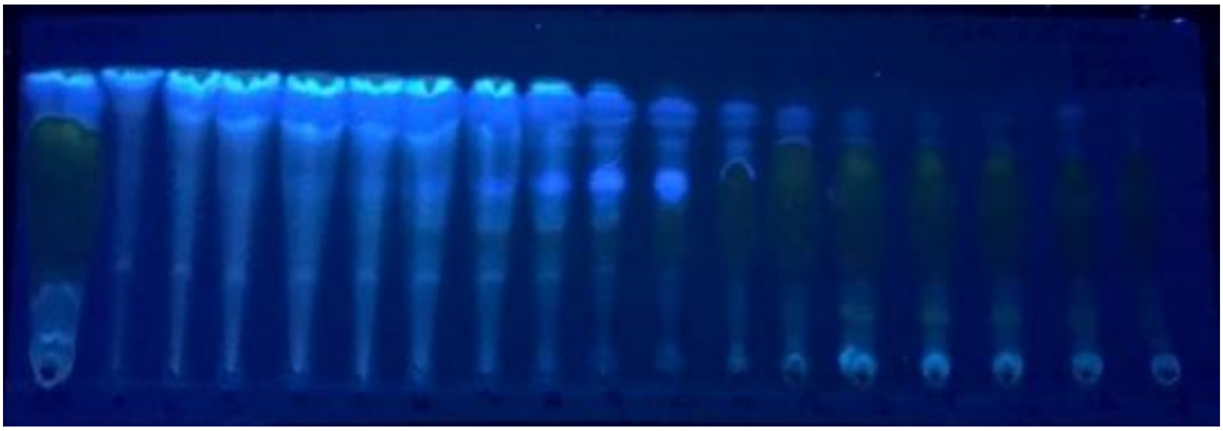

255 nm

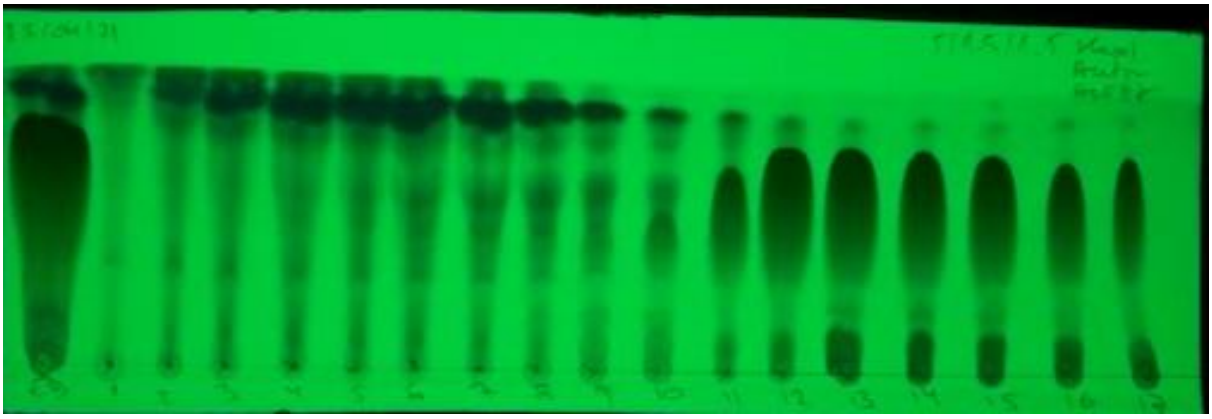

After vanillin solution reagent

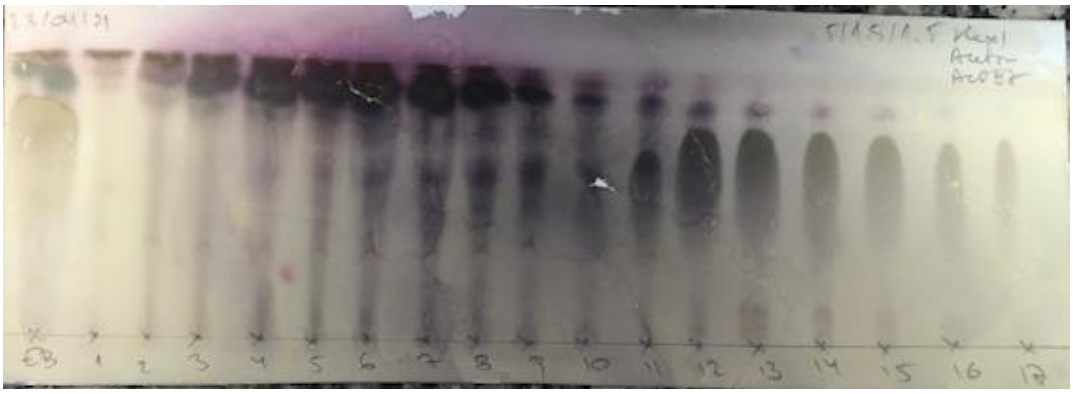

**Continuation**

366 nm

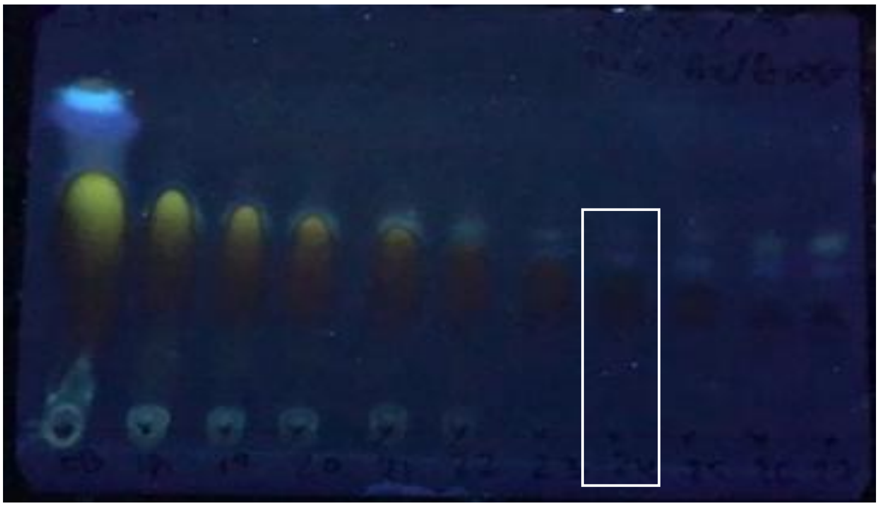

254 nm

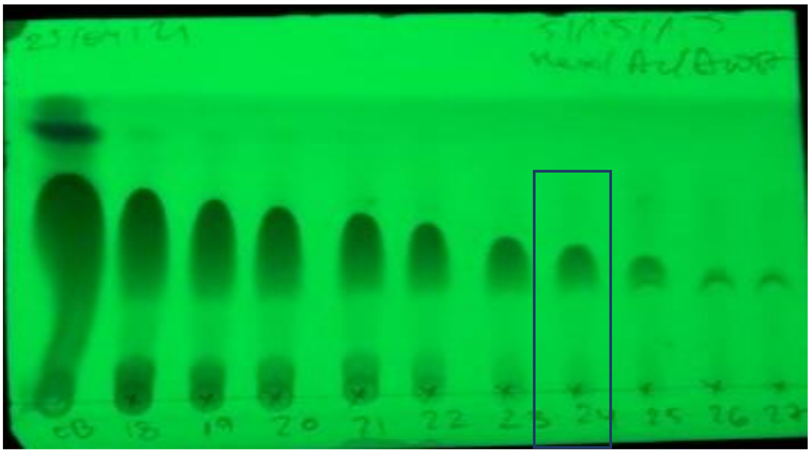

Aftre vanillin revelation

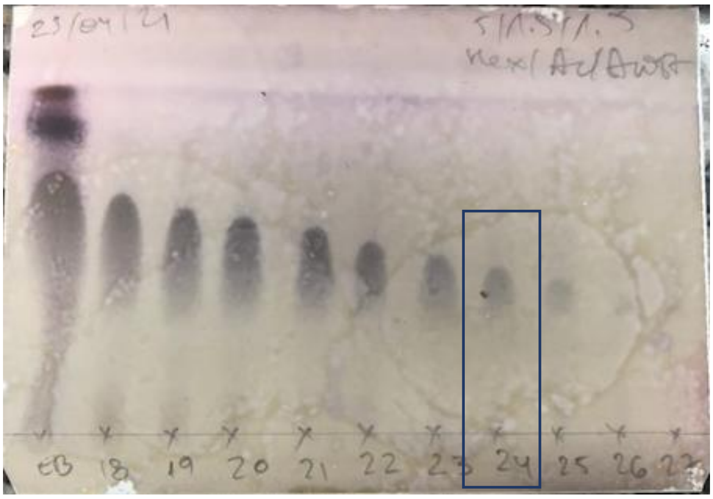

*Figure S2: TLC profiles of CCC first run, from fraction 1 to 27 (separated into two parts), in 366 nm, 254 nm and after TLC visualization reagent, respectively; 5μL application for each fraction, cohumulone is encircled*

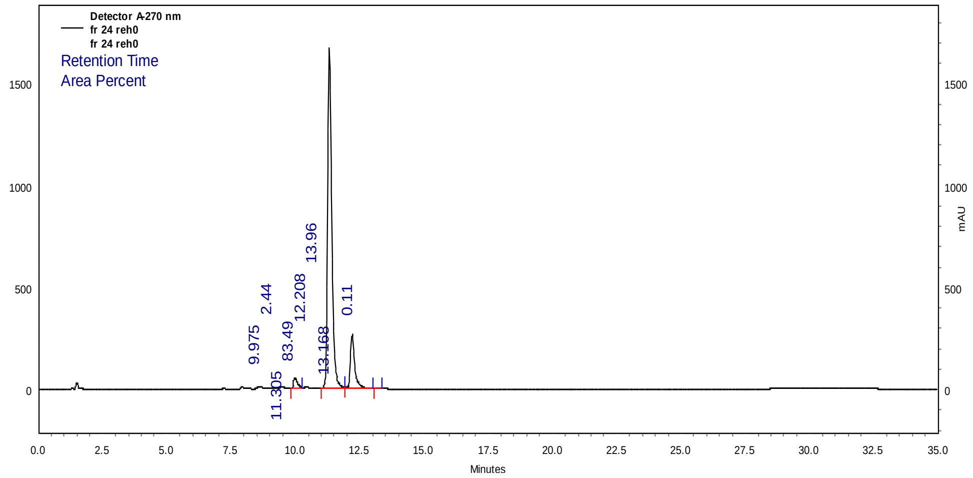


*Figure S3: HPLC profile of fraction 24; 84 % S for cohumulone (25.41% S crude extract)*

**SECOND RUN CCC – TLC & HPLC**

By decreasing slowly the flow rate, a better separation of the two groups acylphloroglucinols could be expected. After HPLC analysis, efficient separation of alfa from beta acids was concluded. Some of the fractions analyzed were prepared to be tested for antiviral activity. The choice was led by keeping either a group of compounds of each group or enriched fractions with a major component.

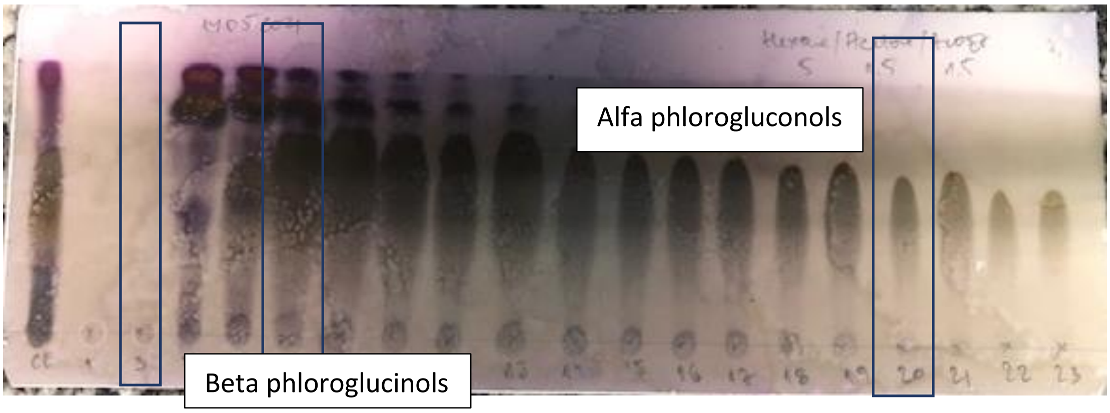

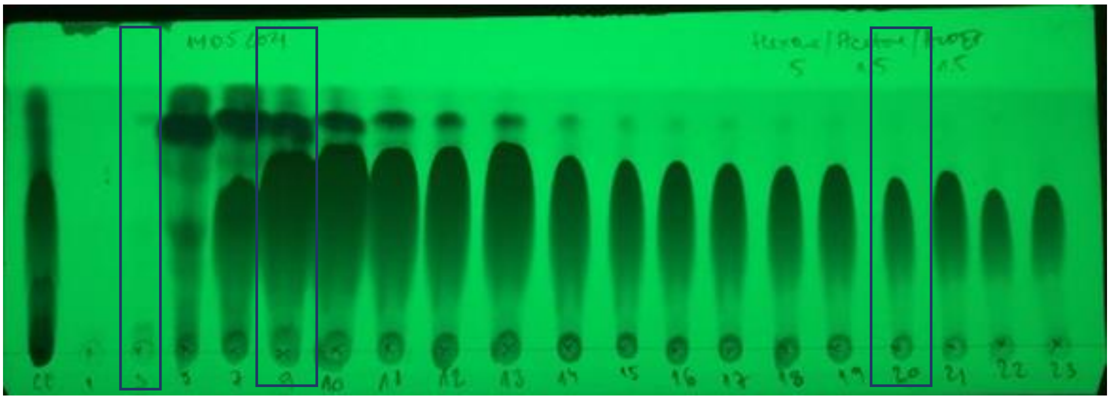

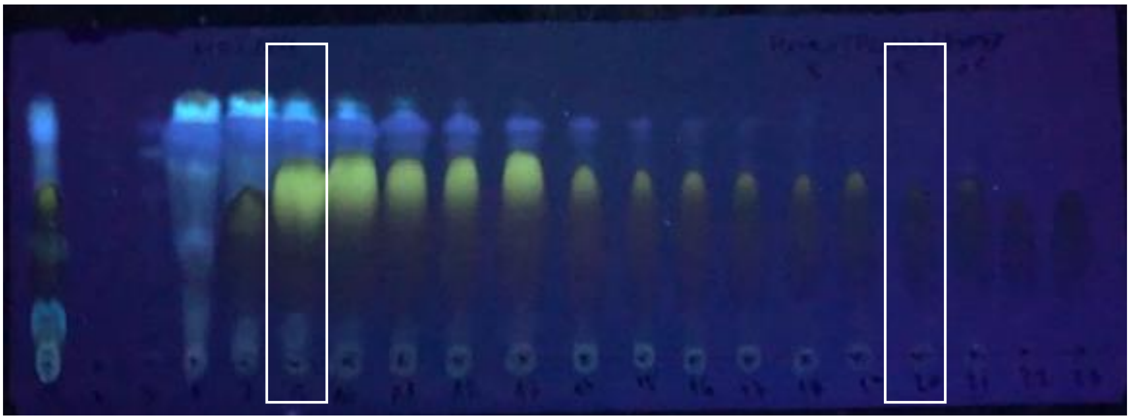

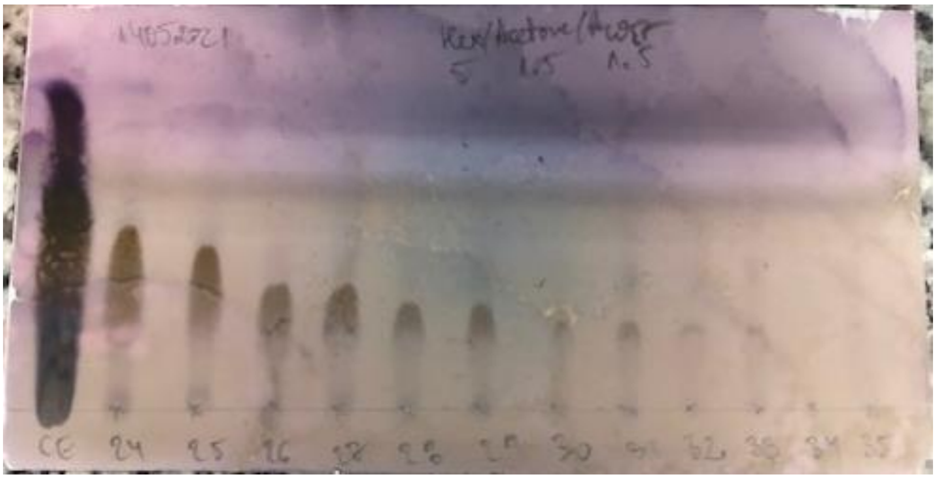

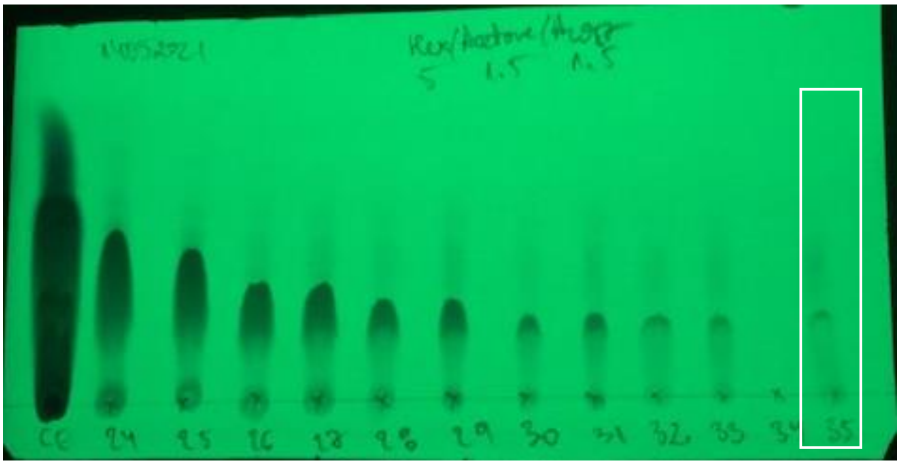

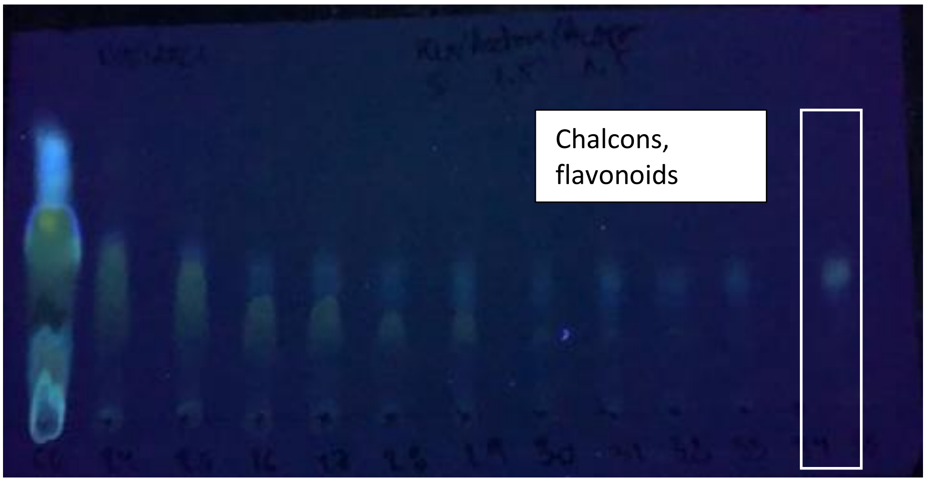


*Figure S4: TLC profiles of CCC second run, from fraction 1 to 35 (separated into two parts), after TLC visualization reagent, 254 nm and 366 nm respectively ; 5µL application for each fraction. All fractions of interest used in the study are encircled*

Thus, 4 fractions for further investigation were retained from the second run:

| fraction | Quantity |
|---|---|
| 35 | 7.3 mg |
| 20 | 13 mg |
| 9 | 80.56 mg |
| 3 | 36.8 mg |

*Table S5 : Quantitative analyses of the selected fractions for further investigation*

Five fractions were retained and chosen by keeping some diversity in the ratio of the congeners from alfa and beta phloroglucinols, besides one fractions enriched on the major chalcone – xanthohumol, after UPLC-MSMS analysis and fitted retention time. The fractions and respective compounds kept to make the *in vitro* tests were defined as follow:

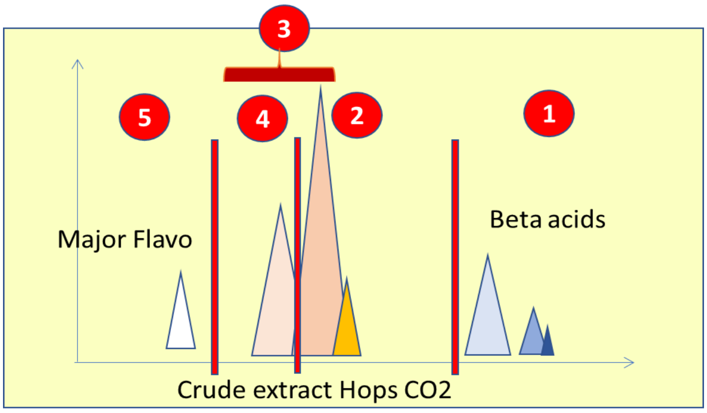


*Figure S5: Schematic representation of the crude $CO_2$ hops extract; red lines indicate each of the fractions selected for further investigation*

1. **Fraction 3** - Beta acids with impurities of alfa (around 5%)
2. **Fraction 9**- Major humulone A2 with 1/3 of cohumulone and adhumulone and 10 % beta acids
3. **Fraction 20** - Only Alfa acids in Ration 1/1/0.5 –A1;A2;A3
4. **Fraction 24** – Major alfa acid - cohumulone A1
5. **Fraction 35** - Major chalcon Xanthohumol

| Number of selected fractions for further investigation | № of the fraction annotated in the text after the HPLC elution methods | Chemical group or compound name |
|---|---|---|
| **1** | **3** | **Beta acids** |
| **2** | 9 | **Alpha and beta acids** |
| **3** | **20** | **Alpha acids** |
| **4** | **24** | **Cohumulone** |
| **5** | **35** | **Xanthohumol** |

*Table S6: Selected fraction after HPLC analysis and their annotation in the manuscript*

**HPLC profiles (purity described as %S) and physicochemical data of the isolated compounds**

Crude extract (CE)

| | Crude Extract | |
|---|---|---|
| | Retention time | Purity %S |
| Xanthohumol | **9.95** | *0.5* |
| Cohumulone | **11.29** | *25.41* |
| Humulone | **12.13** | *41.34* |
| Adhumulone | **12.39** | *11.46* |
| Colupulone | **14.86** | *9.78* |
| Lupulone | **16.01** | *5.67* |
| Adlupulone | **16.27** | *1.72* |

*Table S7: HPLC retention times and purity (%S) of all analysed compounds in the crude $CO_2$ extract*

Fraction 35 – Xanthohumol

| | Purity HPLC | |
|---|---|---|
| | %S CE | % S |
| Xanthohumol | *0.5* | 65,18 |
| Cohumulone | *25.41* | 7,01 |

*Table S8: HPLC purity (%S) of xanthohumol and cohumulone in the crude $CO_2$ extract and in fraction 35, respectively*

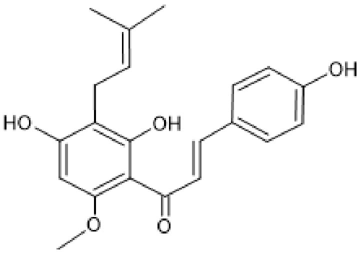


*Figure S6 : Chemical structure of xanthohumol (7)*

Fraction 24 - Cohumulone

| | Purity | |
|---|---|---|
| | %S CE | % S |
| Xanthohumol | *0.5* | 2,44 |
| Cohumulone | *25.41* | 83,49 |
| Humulone | *41.34* | 13,96 |

*Table S9: HPLC purity (%S) of xanthohumol, cohumulone and humulone in the crude $CO_2$ extract and in fraction 24, respectively*

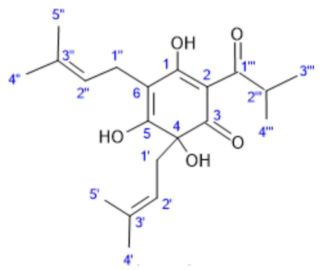


*Figure S7: Chemical structure of cohumulone (5)*

| Carbon atom | | δ¹ H | COSY | δ¹ C HSQC | HMBC |
|---|---|---|---|---|---|
| 1 | | / | | / | 176.44 |
| 2 | | / | | / | 117.24 |
| 3 | | / | | / | 206 |
| 4 | | / | | / | 115.84 |
| 5 | | / | | / | 205.81 |
| 6 | | / | | / | 89.28 |
| 1′ | Ha | 2.54 | | 42.6 | / |
| | Hb | 2.54 | | | / |
| 2′ | | 5.00 | 1.77 ; 2.54 | 116.3 | / |
| 3′ | | / | | 132.86 | / |
| 4′ | 3H | 1.73 | | 25.64 | / |
| 5′ | 3H | 1.13 | 1.53 ;1.77 ; 3.71 | 19.76 | / |
| 1′′ | Ha | 3.07 | 1.69 ; 1.74 ; 5.13 | 21.68 | / |
| | Hb | 3.07 | | | / |
| 2′′ | | 5.13 | 1.73 ; 3.10 | 121.79 | / |
| 3′′ | | / | | 138.3 | / |
| 4′′ | 3H | 1.69 | 1.56 ; 2.06 | 25.77 | / |
| 5′′ | 3H | 1.69 | | 25.77 | / |
| 1′′′ | | / | | 217 | / |
| 2′′′ | | 3.71 | 1.13 ; 1.21 | 35.07 | / |
| 3′′′ | 3H | 1.21 | 1.77 ; 3.71 | 19.76 | / |
| 4′′′ | 3H | 1.53 | 2.13 | 18.66 | / |

*Table S10: 1H (400 MHz) and 13C (300 MHz) NMR data of cohumulone in chloroform D*

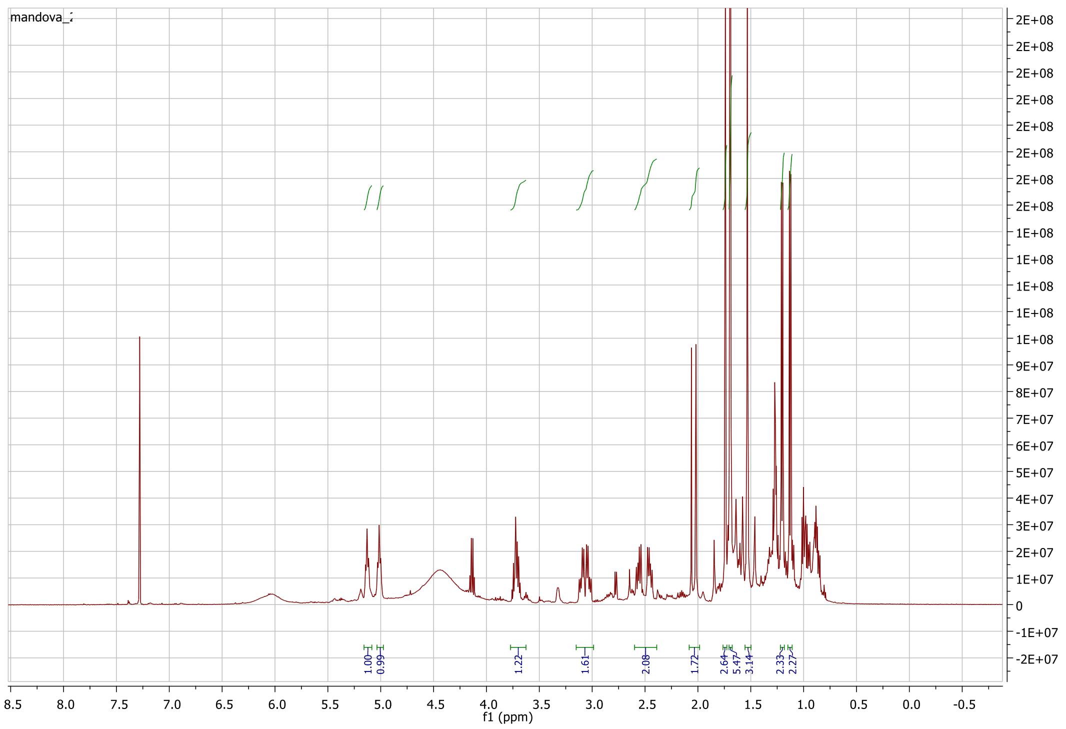

*Figure S8 : 1H NMR spectrum of cohumulone (5) (400 MHz, chloroform D)*

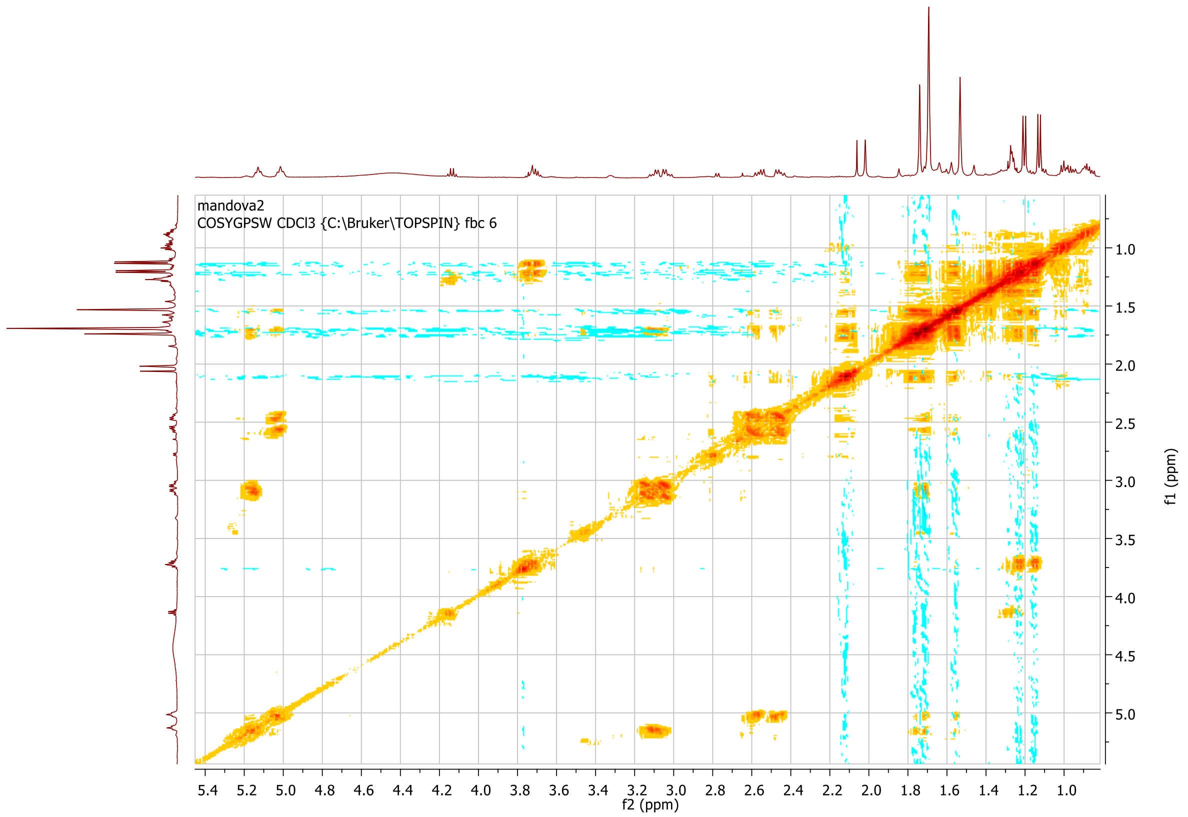

*Figure S9: 13C NMR COSYgp spectrum of cohumulone (5) (300 MHz, chloroform D)*

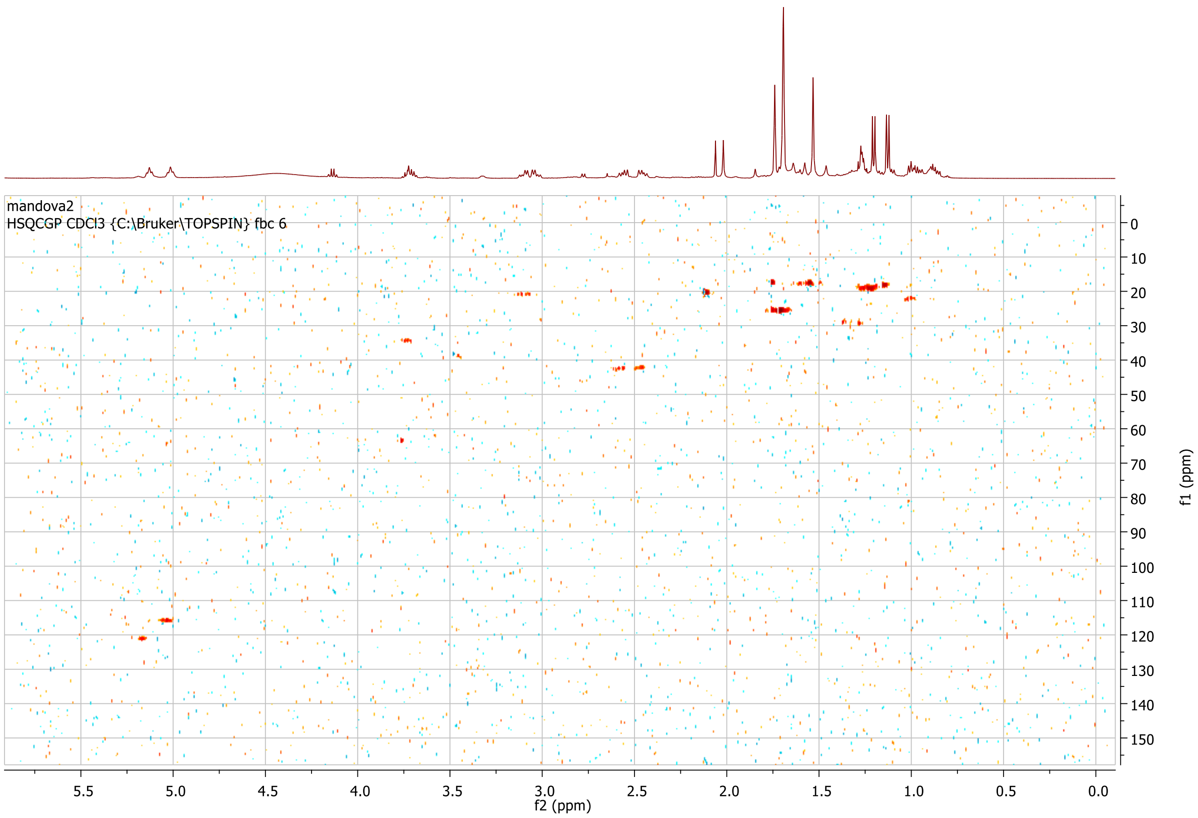


*Figure S10: 13C NMR HSQC spectrum of cohumulone (5) (300 MHz, chloroform D)*

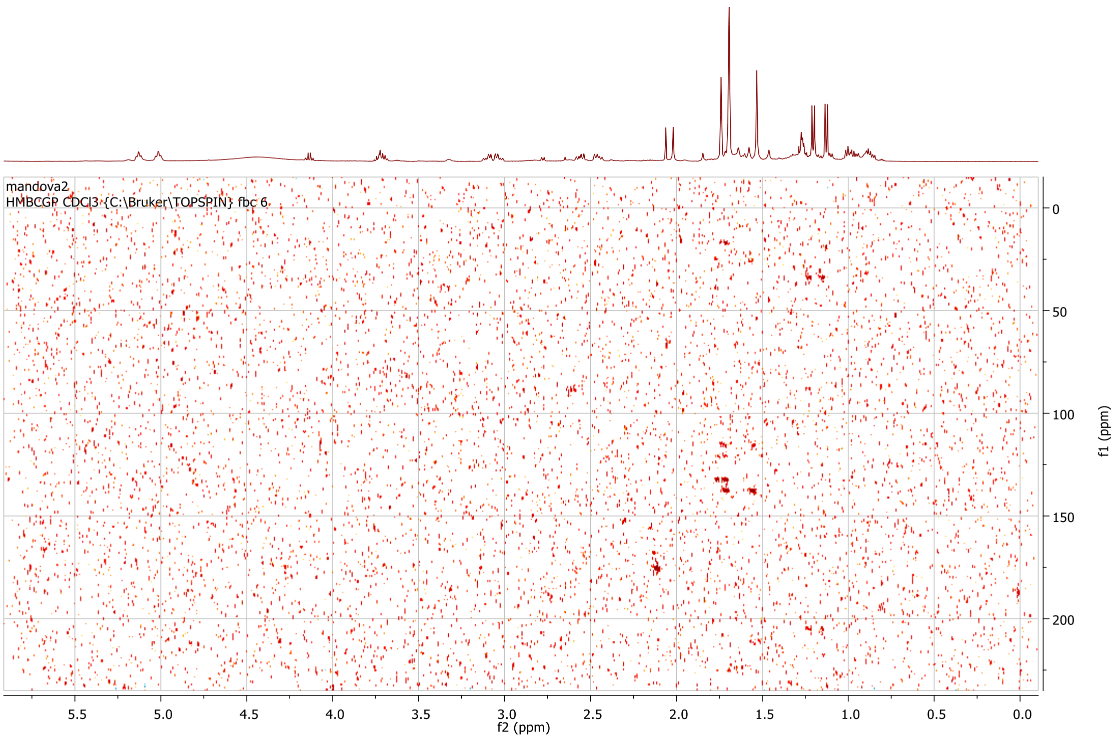


*Figure S11: 13C NMR HMBC spectrum of cohumulone (5) (300 MHz, chloroform D)*

Fraction 20 - Alpha acids

| | Purity | |
|---|---|---|
| | %S CE | % S |
| Cohumulone | *25.41* | 45,82 |
| Humulone | *41.34* | 46,67 |
| Adhumulone | *11.46* | 6,96 |

*Table S11 : HPLC purity (%S) of cohumulone, humulone and adhumulone in the crude $CO_2$ extract and in fraction 20, respectively*

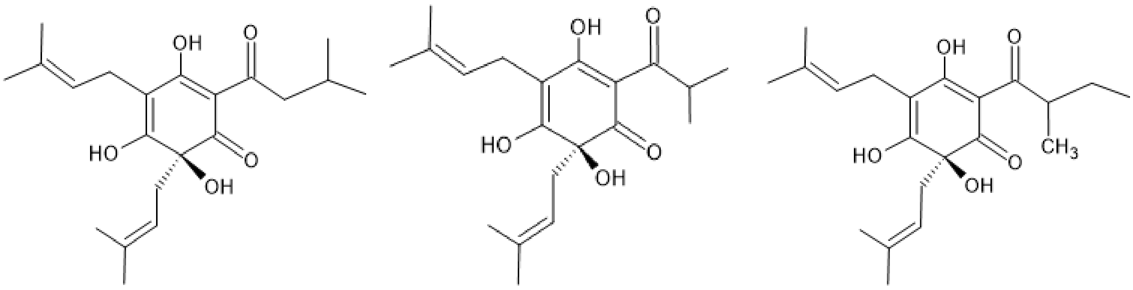


humulone (4) cohumulone (5) adhumulone (6)

*Figure S12: Chemical structure of alpha acids*

Fraction 9 - Alpha and beta acids

| | Purity | |
|---|---|---|
| | %S CE | % S |
| Cohumulone | *25.41* | 15,61 |
| Humulone | *41.34* | 49,59 |
| Adhumulone | *11.46* | 17,98 |
| Colupulone | *9.78* | 8,01 |
| Lupulone | *5.67* | 3,88 |
| Adlupulone | *1.72* | 1,3 |

*Table S12 : HPLC purity (%S) of all described and studied alpha and beta acids, in the crude $CO_2$ extract and in fraction 9, respectively*

Fraction 3 - Beta acids

| | Purity | |
|---|---|---|
| | %S CE | % S |
| Colupulone | *9.78* | 32,02 |
| Lupulone | *5.67* | 22,5 |
| Adlupulone | *1.72* | 8,71 |

*Table S13: HPLC purity (%S) of colupulone, lupulone and adlupulone, in the crude $CO_2$ extract and in fraction 3, respectively*

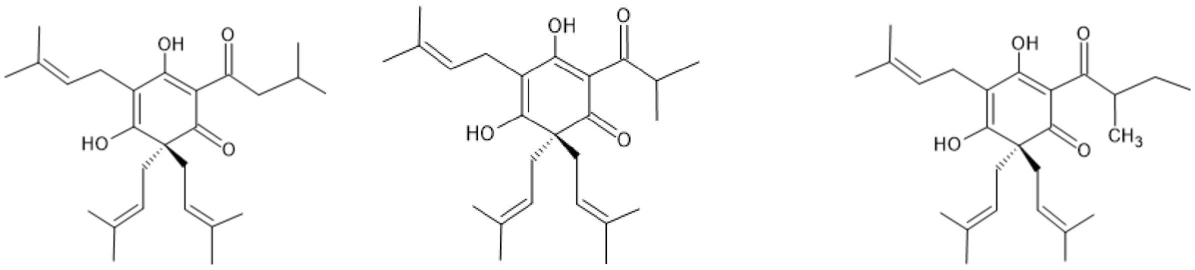

lupulone (1) colupulone (2) adlupulone (3)

*Figure S13: Chemical structure of beta acids*

## Antiviral part

### MTT assay

| | Beta acids | | | | | | | | |
|---|---|---|---|---|---|---|---|---|---|
| | 1st Day | | | 2nd Day | | | 3rd Day | | |
| 250 µg | 80,33869 | 93,30885 | 76,80036 | 103,3571 | 115,8862 | 105,8123 | 117,1379 | 106,6964 | 78,86703 |
| 125 µg | 102,0061 | 110,5351 | 77,06952 | 103,747 | 103,0831 | 105,9809 | 98,6927 | 98,37941 | 81,81885 |
| 62,5 µg | 104,9164 | 102,4659 | 103,2846 | 125,0485 | 106,845 | 106,7027 | 95,33947 | 108,8405 | 110,7937 |
| 31,25 µg | 102,9257 | 99,43224 | 84,9032 | 126,1075 | 101,8239 | 102,0715 | 105,2621 | 89,96451 | 74,19208 |
| mean | 97,57831 | | | | | | | | |
| standard deviation | 15,80884 | | | | | | | | |

| | Alpha and beta acids | | | | | | | | |
|---|---|---|---|---|---|---|---|---|---|
| | 1st Day | | | 2nd Day | | | 3rd Day | | |
| 250 µg | 114,7856 | 99,53878 | 89,26023 | 102,0346 | 83,29894 | 78,95222 | 95,93669 | 85,13292 | 108,9237 |
| 125 µg | 105,9089 | 90,35369 | 97,5369 | 111,9135 | 95,56986 | 98,70477 | 82,36222 | 89,1519 | 96,88147 |
| 62,5 µg | 98,86028 | 96,40419 | 89,52378 | 111,2022 | 102,4034 | 96,19684 | 94,61008 | 84,88326 | 103,5635 |
| 31,25 µg | 97,41354 | 101,7145 | 90,83033 | 97,50349 | 95,33803 | 109,295 | 95,69682 | 83,11119 | 90,4834 |
| mean | 95,31819 | | | | | | | | |
| standard deviation | 12,14569 | | | | | | | | |

| | Alpha acids | | | | | | | | |
|---|---|---|---|---|---|---|---|---|---|
| | 1st Day | | | 2nd Day | | | 3rd Day | | |
| 250 µg | 86,19853 | 74,57979 | 87,1462 | 116,8978 | 94,5003 | 79,53178 | 93,01424 | 92,72542 | 85,53922 |
| 125 µg | 99,91449 | 100,8678 | 97,94064 | 98,69423 | 108,0463 | 91,23895 | 93,92475 | 81,85801 | 85,16229 |
| 62,5 µg | 98,73691 | 96,94251 | 94,04904 | 101,8766 | 93,08301 | 78,96803 | 112,3504 | 84,06086 | 115,1798 |
| 31,25 µg | 97,09952 | 94,89577 | 99,84159 | 81,31789 | 87,59297 | 95,29588 | 66,40377 | 99,60811 | 83,45386 |
| mean | 90,01481 | | | | | | | | |
| standard deviation | 11,99017 | | | | | | | | |

| | Cohumulone | | | | | | | | |
|---|---|---|---|---|---|---|---|---|---|
| | 1st Day | | | 2nd Day | | | 3rd Day | | |
| 250 µg | 87,33686 | 98,74252 | 102,1014 | 95,15363 | 99,33175 | 99,8955 | 82,8958 | 87,03716 | 66,24223 |
| 125 µg | 90,59482 | 95,84344 | 105,9426 | 87,41383 | 98,67315 | 94,86912 | 85,77419 | 74,43195 | 77,37398 |
| 62,5 µg | 62,26571 | 96,09578 | 120,7688 | 64,43681 | 93,96816 | 89,79004 | 32,90083 | 91,66805 | 64,3282 |
| 31,25 µg | 90,5051 | 141,0455 | 108,965 | 82,67196 | 100,0904 | 93,08301 | 87,86935 | 92,3338 | 88,6428 |
| mean | 90,97076 | | | | | | | | |
| standard deviation | 11,49309 | | | | | | | | |

| | Xanthohumol | | | | | | | | |
|---|---|---|---|---|---|---|---|---|---|
| | 1st Day | | | 2nd Day | | | 3rd Day | | |
| 250 µg | 98,91074 | 89,39481 | 81,51627 | 106,629 | 113,5837 | 99,07358 | 75,63128 | 101,9578 | 100,7634 |
| 125 µg | 110,3276 | 93,47707 | 96,42662 | 112,6353 | 83,60453 | 99,12627 | 107,1321 | 88,19244 | 86,08749 |
| 62,5 µg | 110,6921 | 97,58176 | 93,6509 | 114,9799 | 110,6332 | 114,2054 | 100,5333 | 112,835 | 68,13179 |
| 31,25 µg | 107,5855 | 117,651 | 102,8528 | 125,8388 | 112,4615 | 86,81847 | 107,602 | 94,72267 | 104,3124 |
| mean | 96,38451 | | | | | | | | |
| standard deviation | 12,0687 | | | | | | | | |

**Plaque reduction test results**

**Virucidal activity**

Number of formed viral plaques by well, for each of the tested concentrations

| | Beta acids | | | | | | | | |
|---|---|---|---|---|---|---|---|---|---|
| | 1st Day | | | 2nd Day | | | 3rd Day | | |
| 125 µg | 4 | 3 | 3 | 4 | 4 | 6 | 4 | 2 | 2 |
| 62,5 µg | 12 | 9 | 9 | 11 | 11 | 12 | 15 | 15 | 15 |
| 31,25 µg | 14 | 24 | 13 | 21 | 19 | 16 | 19 | 19 | 24 |
| control | 70 | 70 | 70 | 70 | 70 | 70 | 70 | 70 | 70 |

-inhibition percentage – relative plate numbers

| | Beta acids | | | | | | | | |
|---|---|---|---|---|---|---|---|---|---|
| | 1st Day | | | 2nd Day | | | 3rd Day | | |
| 125 µg | 94,28571 | 95,71429 | 95,71429 | 94,28571 | 94,28571 | 91,42857 | 94,28571 | 97,14286 | 97,14286 |
| 62,5 µg | 82,85714 | 87,14286 | 87,14286 | 84,28571 | 84,28571 | 82,85714 | 78,57143 | 78,57143 | 78,57143 |
| 31,25 µg | 80 | 65,71429 | 81,42857 | 70 | 72,85714 | 77,14286 | 72,85714 | 72,85714 | 65,71429 |

Number of formed viral plaques by well, for each of the tested concentrations

| | Alpha and beta acids | | | | | | | | |
|---|---|---|---|---|---|---|---|---|---|
| | 1st Day | | | 2nd Day | | | 3rd Day | | |
| 125 µg | 17 | 15 | 15 | 10 | 15 | 15 | 10 | 10 | 14 |
| 62,5 µg | 25 | 24 | 22 | 26 | 26 | 24 | 24 | 28 | 28 |
| 31,25 µg | 32 | 34 | 33 | 30 | 32 | 30 | 27 | 34 | 30 |
| control | 70 | 70 | 70 | 70 | 70 | 70 | 70 | 70 | 70 |

-inhibition percentage – relative plate numbers

| | Alpha and beta acids | | | | | | | | |
|---|---|---|---|---|---|---|---|---|---|
| | 1st Day | | | 2nd Day | | | 3rd Day | | |
| 125 µg | 75,71429 | 78,57143 | 78,57143 | 85,71429 | 78,57143 | 78,57143 | 85,71429 | 85,71429 | 80 |
| 62,5 µg | 64,28571 | 65,71429 | 68,57143 | 62,85714 | 62,85714 | 65,71429 | 65,71429 | 60 | 60 |
| 31,25 µg | 54,28571 | 51,42857 | 52,85714 | 57,14286 | 54,28571 | 57,14286 | 61,42857 | 51,42857 | 57,14286 |

Number of formed viral plaques by well, for each of the tested concentrations

| | Alpha acids | | | | | | | | |
|---|---|---|---|---|---|---|---|---|---|
| | 1st Day | | | 2nd Day | | | 3rd Day | | |
| 125 µg | 12 | 13 | 17 | 16 | 14 | 17 | 12 | 14 | 14 |
| 62,5 µg | 26 | 26 | 26 | 27 | 25 | 25 | 26 | 25 | 27 |
| 31,25 µg | 32 | 33 | 33 | 33 | 32 | 34 | 32 | 33 | 33 |
| control | 70 | 70 | 70 | 70 | 70 | 70 | 70 | 70 | 70 |

-inhibition percentage – relative plate numbers

| | Alpha acids | | | | | | | | |
|---|---|---|---|---|---|---|---|---|---|
| | 1st Day | | | 2nd Day | | | 3rd Day | | |
| 125 µg | 82,85714 | 81,42857 | 75,71429 | 77,14286 | 80 | 75,71429 | 82,85714 | 80 | 80 |
| 62,5 µg | 62,85714 | 62,85714 | 62,85714 | 61,42857 | 64,28571 | 64,28571 | 62,85714 | 64,28571 | 61,42857 |
| 31,25 µg | 54,28571 | 52,85714 | 52,85714 | 52,85714 | 54,28571 | 51,42857 | 54,28571 | 52,85714 | 52,85714 |

Number of formed viral plaques by well, for each of the tested concentrations

| | Cohumulone | | | | | | | | |
|---|---|---|---|---|---|---|---|---|---|
| | 1st Day | | | 2nd Day | | | 3rd Day | | |
| 125 µg | 10 | 12 | 11 | 10 | 10 | 13 | 10 | 20 | 20 |
| 62,5 µg | 22 | 22 | 24 | 26 | 24 | 24 | 25 | 25 | 25 |
| 31,25 µg | 33 | 34 | 33 | 32 | 31 | 31 | 30 | 30 | 31 |
| control | 70 | 70 | 70 | 70 | 70 | 70 | 70 | 70 | 70 |

-inhibition percentage – relative plate numbers

| | Cohumulone | | | | | | | | |
|---|---|---|---|---|---|---|---|---|---|
| | 1st Day | | | 2nd Day | | | 3rd Day | | |
| 125 µg | 85,71429 | 82,85714 | 84,28571 | 85,71429 | 85,71429 | 81,42857 | 85,71429 | 71,42857 | 71,42857 |
| 62,5 µg | 68,57143 | 68,57143 | 65,71429 | 62,85714 | 65,71429 | 65,71429 | 64,28571 | 64,28571 | 64,28571 |
| 31,25 µg | 52,85714 | 51,42857 | 52,85714 | 54,28571 | 55,71429 | 55,71429 | 57,14286 | 57,14286 | 55,71429 |

Number of formed viral plaques by well, for each of the tested concentrations

| | Xanthohumol | | | | | | | | |
|---|---|---|---|---|---|---|---|---|---|
| | 1st Day | | | 2nd Day | | | 3rd Day | | |

| | | | | | | | | | |
|---|---|---|---|---|---|---|---|---|---|
| 125 µg | 18 | 18 | 14 | 15 | 13 | 13 | 14 | 18 | 18 |
| 62,5 µg | 26 | 23 | 23 | 24 | 24 | 26 | 23 | 24 | 24 |
| 31,25 µg | 32 | 32 | 34 | 32 | 32 | 32 | 30 | 32 | 30 |
| control | 70 | 70 | 70 | 70 | 70 | 70 | 70 | 70 | 70 |

-inhibition percentage – relative plate numbers

| | Xanthohumol | | | | | | | | |
|---|---|---|---|---|---|---|---|---|---|
| | 1st Day | | | 2nd Day | | | 3rd Day | | |
| 125 µg | 74,28571 | 74,28571 | 80 | 78,57143 | 81,42857 | 81,42857 | 80 | 74,28571 | 74,28571 |
| 62,5 µg | 62,85714 | 67,14286 | 67,14286 | 65,71429 | 65,71429 | 62,85714 | 67,14286 | 65,71429 | 65,71429 |
| 31,25 µg | 54,28571 | 54,28571 | 51,42857 | 54,28571 | 54,28571 | 54,28571 | 57,14286 | 54,28571 | 57,14286 |

**Co treatment**

Number of formed viral plaques by well, for each of the tested concentrations

| | Beta acids | | | | | | | | |
|---|---|---|---|---|---|---|---|---|---|
| | 1st Day | | | 2nd Day | | | 3rd Day | | |
| 125 µg | 18 | 17 | 22 | 18 | 17 | 28 | 18 | 20 | 20 |
| 62,5 µg | 27 | 32 | 33 | 37 | 34 | 34 | 34 | 32 | 34 |
| 31,25 µg | 50 | 46 | 47 | 49 | 49 | 52 | 47 | 49 | 47 |
| control | 70 | 68 | 60 | 64 | 69 | 64 | 60 | 62 | 54 |

-inhibition percentage – relative plate numbers

| | Beta acids | | | | | | | | |
|---|---|---|---|---|---|---|---|---|---|
| | 1st Day | | | 2nd Day | | | 3rd Day | | |
| 125 µg | 72,72727 | 74,24242 | 66,66667 | 72,58883 | 74,11168 | 57,36041 | 69,31818 | 65,90909 | 65,90909 |
| 62,5 µg | 59,09091 | 51,51515 | 50 | 43,65482 | 48,22335 | 48,22335 | 42,04545 | 45,45455 | 42,04545 |
| 31,25 µg | 24,24242 | 30,30303 | 28,78788 | 25,38071 | 25,38071 | 20,81218 | 19,88636 | 16,47727 | 19,88636 |

Number of formed viral plaques by well, for each of the tested concentrations

| | Alpha and beta acids | | | | | | | | |
|---|---|---|---|---|---|---|---|---|---|
| | 1st Day | | | 2nd Day | | | 3rd Day | | |
| 125 µg | 25 | 26 | 29 | 30 | 27 | 29 | 35 | 34 | 33 |
| 62,5 µg | 38 | 40 | 43 | 41 | 46 | 39 | 40 | 41 | 41 |

| | | | | | | | | | |
|---|---|---|---|---|---|---|---|---|---|
| 31,25 µg | 55 | 52 | 51 | 50 | 52 | 53 | 50 | 48 | 48 |
| control | 70 | 68 | 60 | 64 | 69 | 64 | 60 | 62 | 54 |

-inhibition percentage – relative plate numbers

| | Alpha and beta acids | | | | | | | | |
|---|---|---|---|---|---|---|---|---|---|
| | 1st Day | | | 2nd Day | | | 3rd Day | | |
| 125 µg | 62,12121 | 60,60606 | 56,06061 | 54,31472 | 58,88325 | 55,83756 | 40,34091 | 42,04545 | 43,75 |
| 62,5 µg | 42,42424 | 39,39394 | 34,84848 | 37,56345 | 29,94924 | 40,60914 | 31,81818 | 30,11364 | 30,11364 |
| 31,25 µg | 16,66667 | 21,21212 | 22,72727 | 23,85787 | 20,81218 | 19,28934 | 14,77273 | 18,18182 | 18,18182 |

Number of formed viral plaques by well, for each of the tested concentrations

| | Alpha acids | | | | | | | | |
|---|---|---|---|---|---|---|---|---|---|
| | 1st Day | | | 2nd Day | | | 3rd Day | | |
| 125 µg | 32 | 27 | 28 | 30 | 31 | 27 | 27 | 29 | 29 |
| 62,5 µg | 42 | 42 | 48 | 43 | 41 | 41 | 43 | 43 | 45 |
| 31,25 µg | 55 | 50 | 52 | 52 | 50 | 51 | 47 | 50 | 49 |
| control | 70 | 68 | 60 | 64 | 69 | 64 | 60 | 62 | 54 |

-inhibition percentage – relative plate numbers

| | Alpha acids | | | | | | | | |
|---|---|---|---|---|---|---|---|---|---|
| | 1st Day | | | 2nd Day | | | 3rd Day | | |
| 125 µg | 51,51515 | 59,09091 | 57,57576 | 54,31472 | 52,79188 | 58,88325 | 53,97727 | 50,56818 | 50,56818 |
| 62,5 µg | 36,36364 | 36,36364 | 27,27273 | 34,51777 | 37,56345 | 37,56345 | 26,70455 | 26,70455 | 23,29545 |
| 31,25 µg | 16,66667 | 24,24242 | 21,21212 | 20,81218 | 23,85787 | 22,33503 | 19,88636 | 14,77273 | 16,47727 |

Number of formed viral plaques by well, for each of the tested concentrations

| | Cohumulone | | | | | | | | |
|---|---|---|---|---|---|---|---|---|---|
| | 1st Day | | | 2nd Day | | | 3rd Day | | |
| 125 µg | 36 | 32 | 30 | 33 | 32 | 35 | 36 | 36 | 34 |
| 62,5 µg | 42 | 38 | 40 | 41 | 41 | 47 | 40 | 42 | 38 |
| 31,25 µg | 46 | 43 | 48 | 50 | 49 | 52 | 49 | 49 | 45 |
| control | 70 | 68 | 60 | 64 | 69 | 64 | 60 | 62 | 54 |

-inhibition percentage – relative plate numbers

| | Cohumulone | | | | | | | | |
|---|---|---|---|---|---|---|---|---|---|
| | 1st Day | | | 2nd Day | | | 3rd Day | | |
| 125 µg | 45,45455 | 51,51515 | 54,54545 | 49,74619 | 51,26904 | 46,70051 | 38,63636 | 38,63636 | 42,04545 |
| 62,5 µg | 36,36364 | 42,42424 | 39,39394 | 37,56345 | 37,56345 | 28,4264 | 31,81818 | 28,40909 | 35,22727 |
| 31,25 µg | 30,30303 | 34,84848 | 27,27273 | 23,85787 | 25,38071 | 20,81218 | 16,47727 | 16,47727 | 23,29545 |

Number of formed viral plaques by well, for each of the tested concentrations

| | Xanthohumol | | | | | | | | |
|---|---|---|---|---|---|---|---|---|---|
| | 1st Day | | | 2nd Day | | | 3rd Day | | |
| 125 µg | 21 | 27 | 27 | 36 | 40 | 40 | 39 | 39 | 37 |
| 62,5 µg | 42 | 39 | 39 | 47 | 45 | 43 | 45 | 45 | 46 |
| 31,25 µg | 52 | 53 | 51 | 52 | 51 | 45 | 50 | 50 | 49 |
| control | 70 | 68 | 60 | 64 | 69 | 64 | 60 | 62 | 54 |

-inhibition percentage – relative plate numbers

| | Xanthohumol | | | | | | | | |
|---|---|---|---|---|---|---|---|---|---|
| | 1st Day | | | 2nd Day | | | 3rd Day | | |
| 125 µg | 68,18182 | 59,09091 | 59,09091 | 45,17766 | 39,08629 | 39,08629 | 33,52273 | 33,52273 | 36,93182 |
| 62,5 µg | 36,36364 | 40,90909 | 40,90909 | 28,4264 | 31,47208 | 34,51777 | 23,29545 | 23,29545 | 21,59091 |
| 31,25 µg | 21,21212 | 19,69697 | 22,72727 | 20,81218 | 22,33503 | 31,47208 | 14,77273 | 14,77273 | 16,47727 |

**Post treatment**

Number of formed viral plaques by well, for each of the tested concentrations

| | Beta acids | | | | | | | | |
|---|---|---|---|---|---|---|---|---|---|
| | 1st Day | | | 2nd Day | | | 3rd Day | | |
| 125 µg | 11 | 14 | 14 | 14 | 13 | 13 | 13 | 13 | 14 |
| 62,5 µg | 47 | 51 | 47 | 44 | 37 | 44 | 33 | 39 | 40 |
| 31,25 µg | 54 | 54 | 54 | 55 | 59 | 58 | 52 | 52 | 55 |
| control | 56 | 56 | 54 | 50 | 66 | 62 | 57 | 51 | 55 |

-inhibition percentage – relative plate numbers

| | Beta acids | | | | | | | | |
|---|---|---|---|---|---|---|---|---|---|
| | 1st Day | | | 2nd Day | | | 3rd Day | | |
| 125 µg | 80,12048 | 74,6988 | 74,6988 | 76,40449 | 78,08989 | 78,08989 | 76,07362 | 76,07362 | 74,23313 |
| 62,5 µg | 15,06024 | 7,831325 | 15,06024 | 25,8427 | 37,64045 | 25,8427 | 39,2638 | 28,22086 | 26,38037 |
| 31,25 µg | 2,409639 | 2,409639 | 2,409639 | 7,303371 | 0,561798 | 2,247191 | 4,294479 | 4,294479 | -1,22699 |

Number of formed viral plaques by well, for each of the tested concentrations

| | Alpha and beta acids | | | | | | | | |
|---|---|---|---|---|---|---|---|---|---|
| | 1st Day | | | 2nd Day | | | 3rd Day | | |
| 125 µg | 32 | 35 | 35 | 32 | 33 | 35 | 36 | 34 | 33 |
| 62,5 µg | 45 | 49 | 44 | 59 | 59 | 59 | 53 | 54 | 54 |
| 31,25 µg | 55 | 52 | 51 | 59 | 59 | 61 | 52 | 54 | 54 |
| control | 56 | 56 | 54 | 50 | 66 | 62 | 57 | 51 | 55 |

-inhibition percentage – relative plate numbers

| | Alpha and beta acids | | | | | | | | |
|---|---|---|---|---|---|---|---|---|---|
| | 1st Day | | | 2nd Day | | | 3rd Day | | |
| 125 µg | 42,16867 | 36,74699 | 36,74699 | 46,06742 | 44,38202 | 41,01124 | 33,74233 | 37,42331 | 39,2638 |
| 62,5 µg | 18,6747 | 11,44578 | 20,48193 | 0,561798 | 0,561798 | 0,561798 | 2,453988 | 0,613497 | 0,613497 |
| 31,25 µg | 0,60241 | 6,024096 | 7,831325 | 0,561798 | 0,561798 | -2,80899 | 4,294479 | 0,613497 | 0,613497 |

Number of formed viral plaques by well, for each of the tested concentrations

| | Alpha acids | | | | | | | | |
|---|---|---|---|---|---|---|---|---|---|
| | 1st Day | | | 2nd Day | | | 3rd Day | | |
| 125 µg | 13 | 13 | 13 | 13 | 15 | 15 | 12 | 12 | 13 |
| 62,5 µg | 52 | 52 | 54 | 59 | 58 | 58 | 53 | 55 | 55 |
| 31,25 µg | x | x | x | x | x | x | x | x | x |
| control | 56 | 56 | 54 | 50 | 66 | 62 | 57 | 51 | 55 |

-inhibition percentage – relative plate numbers

| | Alpha acids | | | | | | | | |
|---|---|---|---|---|---|---|---|---|---|
| | 1st Day | | | 2nd Day | | | 3rd Day | | |
| 125 µg | 76,50602 | 76,50602 | 76,50602 | 78,08989 | 74,7191 | 74,7191 | 77,91411 | 77,91411 | 76,07362 |
| 62,5 µg | 6,024096 | 6,024096 | 2,409639 | 0,561798 | 2,247191 | 2,247191 | 2,453988 | -1,22699 | -1,22699 |

| | | | | | | | | | |
|---|---|---|---|---|---|---|---|---|---|
| 31,25 µg | x | x | x | x | x | x | x | x | x |

Number of formed viral plaques by well, for each of the tested concentrations

| | Cohumulone | | | | | | | | |
|---|---|---|---|---|---|---|---|---|---|
| | 1st Day | | | 2nd Day | | | 3rd Day | | |
| 125 µg | 11 | 15 | 15 | 15 | 17 | 17 | 20 | 20 | 19 |
| 62,5 µg | 40 | 41 | 40 | 38 | 37 | 39 | 54 | 54 | 54 |
| 31,25 µg | 55 | 55 | 54 | 58 | 58 | 59 | 56 | 54 | 56 |
| control | 56 | 56 | 54 | 50 | 66 | 62 | 57 | 51 | 55 |

-inhibition percentage – relative plate numbers

| | Cohumulone | | | | | | | | |
|---|---|---|---|---|---|---|---|---|---|
| | 1st Day | | | 2nd Day | | | 3rd Day | | |
| 125 µg | 80,12048 | 72,89157 | 72,89157 | 74,7191 | 71,34831 | 71,34831 | 63,19018 | 63,19018 | 65,03067 |
| 62,5 µg | 27,71084 | 25,90361 | 27,71084 | 35,95506 | 37,64045 | 34,26966 | 0,613497 | 0,613497 | 0,613497 |
| 31,25 µg | 0,60241 | 0,60241 | 2,409639 | 2,247191 | 2,247191 | 0,561798 | -3,06748 | 0,613497 | -3,06748 |

Number of formed viral plaques by well, for each of the tested concentrations

| | Xanthohumol | | | | | | | | |
|---|---|---|---|---|---|---|---|---|---|
| | 1st Day | | | 2nd Day | | | 3rd Day | | |
| 125 µg | 14 | 11 | 11 | 12 | 12 | 17 | 20 | 13 | 20 |
| 62,5 µg | 52 | 55 | 55 | 58 | 58 | 55 | 53 | 53 | 55 |
| 31,25 µg | 55 | 56 | 56 | 59 | 59 | 61 | 56 | 56 | 55 |
| control | 56 | 56 | 54 | 50 | 66 | 62 | 57 | 51 | 55 |

-inhibition percentage – relative plate numbers

| | Xanthohumol | | | | | | | | |
|---|---|---|---|---|---|---|---|---|---|
| | 1st Day | | | 2nd Day | | | 3rd Day | | |
| 125 µg | 74,6988 | 80,12048 | 80,12048 | 79,77528 | 79,77528 | 71,34831 | 63,19018 | 76,07362 | 63,19018 |
| 62,5 µg | 6,024096 | 0,60241 | 0,60241 | 2,247191 | 2,247191 | 7,303371 | 2,453988 | 2,453988 | -1,22699 |
| 31,25 µg | 0,60241 | -1,20482 | -1,20482 | 0,561798 | 0,561798 | -2,80899 | -3,06748 | -3,06748 | -1,22699 |

**Pre treatment**

Number of formed viral plaques by well, for each of the tested concentrations

| | Beta acids | | | | | | | | |
|---|---|---|---|---|---|---|---|---|---|
| | 1st Day | | | 2nd Day | | | 3rd Day | | |
| 125 µg | 36 | 35 | 35 | 38 | 39 | 37 | | | |
| 62,5 µg | 32 | 41 | 41 | 43 | 55 | 50 | | | |
| 31,25 µg | 41 | 40 | 34 | 49 | 52 | 50 | | | |
| control | 43 | 39 | 29 | 51 | 47 | 50 | | | |

-inhibition percentage – relative plate numbers

| | Beta acids | | | | | | | | |
|---|---|---|---|---|---|---|---|---|---|
| | 1st Day | | | 2nd Day | | | 3rd Day | | |
| 125 µg | 2,702703 | 5,405405 | 5,405405 | 22,97297 | 20,94595 | 25 | | | |
| 62,5 µg | 13,51351 | -10,8108 | -10,8108 | 12,83784 | -11,4865 | -1,35135 | | | |
| 31,25 µg | -10,8108 | -8,10811 | 8,108108 | 0,675676 | -5,40541 | -1,35135 | | | |

Number of formed viral plaques by well, for each of the tested concentrations

| | Alpha and beta acids | | | | | | | | |
|---|---|---|---|---|---|---|---|---|---|
| | 1st Day | | | 2nd Day | | | 3rd Day | | |
| 125 µg | 32 | 32 | 37 | 34 | 38 | 46 | | | |
| 62,5 µg | 41 | 41 | 42 | 55 | 46 | 45 | | | |
| 31,25 µg | 42 | 42 | 40 | 48 | 58 | 53 | | | |
| control | 43 | 39 | 29 | 51 | 47 | 50 | | | |

-inhibition percentage – relative plate numbers

| | Alpha and beta acids | | | | | | | | |
|---|---|---|---|---|---|---|---|---|---|
| | 1st Day | | | 2nd Day | | | 3rd Day | | |
| 125 µg | 13,51351 | 13,51351 | 0 | 31,08108 | 22,97297 | 6,756757 | | | |
| 62,5 µg | -10,8108 | -10,8108 | -13,5135 | -11,4865 | 6,756757 | 8,783784 | | | |
| 31,25 µg | -13,5135 | -13,5135 | -8,10811 | 2,702703 | -17,5676 | -7,43243 | | | |

Number of formed viral plaques by well, for each of the tested concentrations

| | Alpha acids | | |
|---|---|---|---|
| | 1st Day | 2nd Day | 3rd Day |

| | | | | | | | | | |
|---|---|---|---|---|---|---|---|---|---|
| 125 µg | 35 | 37 | 34 | 39 | 42 | 32 | | | |
| 62,5 µg | 40 | 41 | 44 | 49 | 49 | 47 | | | |
| 31,25 µg | 40 | 40 | 49 | 50 | 54 | 49 | | | |
| control | 43 | 39 | 29 | 51 | 47 | 50 | | | |

-inhibition percentage – relative plate numbers

| | Alpha acids | | | | | | | | |
|---|---|---|---|---|---|---|---|---|---|
| | 1st Day | | | 2nd Day | | | 3rd Day | | |
| 125 µg | 5,405405 | 0 | 8,108108 | 20,94595 | 14,86486 | 35,13514 | | | |
| 62,5 µg | -8,10811 | -10,8108 | -18,9189 | 0,675676 | 0,675676 | 4,72973 | | | |
| 31,25 µg | -8,10811 | -8,10811 | -32,4324 | -1,35135 | -9,45946 | 0,675676 | | | |

Number of formed viral plaques by well, for each of the tested concentrations

| | Cohumulone | | | | | | | | |
|---|---|---|---|---|---|---|---|---|---|
| | 1st Day | | | 2nd Day | | | 3rd Day | | |
| 125 µg | 40 | 41 | 40 | 48 | 51 | 50 | | | |
| 62,5 µg | 42 | 43 | 42 | 49 | 47 | 47 | | | |
| 31,25 µg | 40 | 42 | 40 | 54 | 53 | 50 | | | |
| control | 43 | 39 | 29 | 51 | 47 | 50 | | | |

-inhibition percentage – relative plate numbers

| | Cohumulone | | | | | | | | |
|---|---|---|---|---|---|---|---|---|---|
| | 1st Day | | | 2nd Day | | | 3rd Day | | |
| 125 µg | - 8,10811 | - 10,8108 | - 8,10811 | 2,702703 | - 3,37838 | - 1,35135 | | | |
| 62,5 µg | - 13,5135 | - 16,2162 | - 13,5135 | 0,675676 | 4,72973 | 4,72973 | | | |
| 31,25 µg | - 8,10811 | - 13,5135 | - 8,10811 | -9,45946 | - 7,43243 | - 1,35135 | | | |

Number of formed viral plaques by well, for each of the tested concentrations

| | Xanthohumol | | |
|---|---|---|---|
| | 1st Day | 2nd Day | 3rd Day |

| | | | | | | | | | |
|---|---|---|---|---|---|---|---|---|---|
| 125 µg | 34 | 40 | 40 | 51 | 54 | 54 | | | |
| 62,5 µg | 42 | 40 | 43 | 50 | 48 | 53 | | | |
| 31,25 µg | 43 | 40 | 43 | 50 | 50 | 54 | | | |
| control | 43 | 39 | 29 | 51 | 47 | 50 | | | |

-inhibition percentage – relative plate numbers

| | Xanthohumol | | | | | | | | |
|---|---|---|---|---|---|---|---|---|---|
| | 1st Day | | | 2nd Day | | | 3rd Day | | |
| 125 µg | 8,108108 | -8,10811 | -8,10811 | -3,37838 | -9,45946 | -9,45946 | | | |
| 62,5 µg | -13,5135 | -8,10811 | -16,2162 | -1,35135 | 2,702703 | -7,43243 | | | |
| 31,25 µg | -16,2162 | -8,10811 | -16,2162 | -1,35135 | -1,35135 | -9,45946 | | | |

**Immunofluorescence – Virucidal activity**

CHIKV was cultured in Vero cells with the compounds of interest at 125 µg/mL. The cells was challenged with MOI=0,1 of virus. Mock and CHIKV-infected cells without compounds were used as controls. The expression of CHIKV Envelope was detected by IFA, with MIAF antibody stained with AlexaFluor 555 antibody.

**Beta acids**

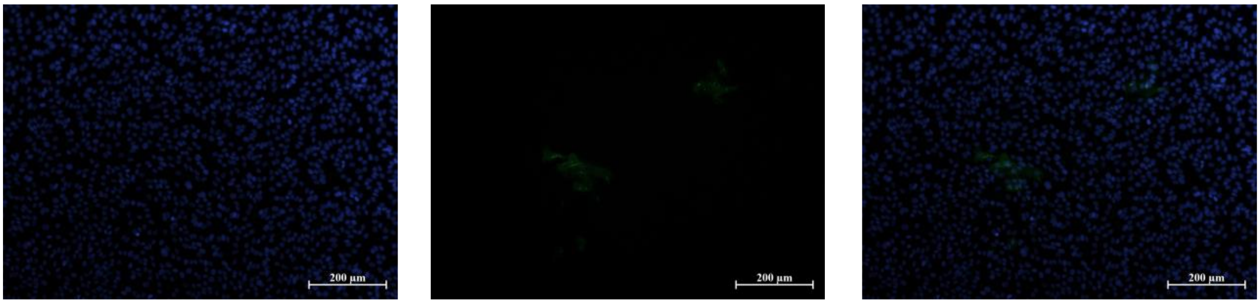


**Alpha and beta acids**

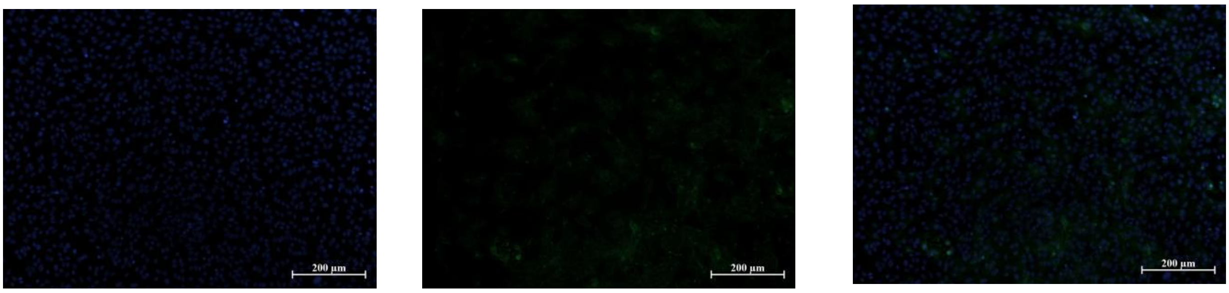

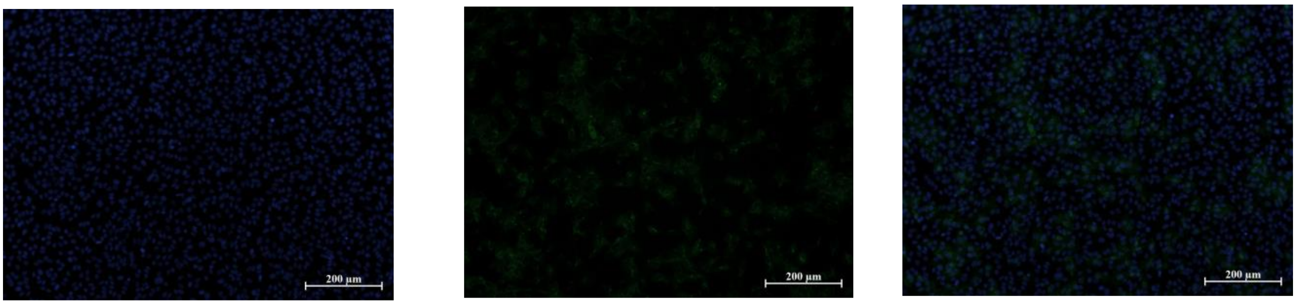


Cohumulone

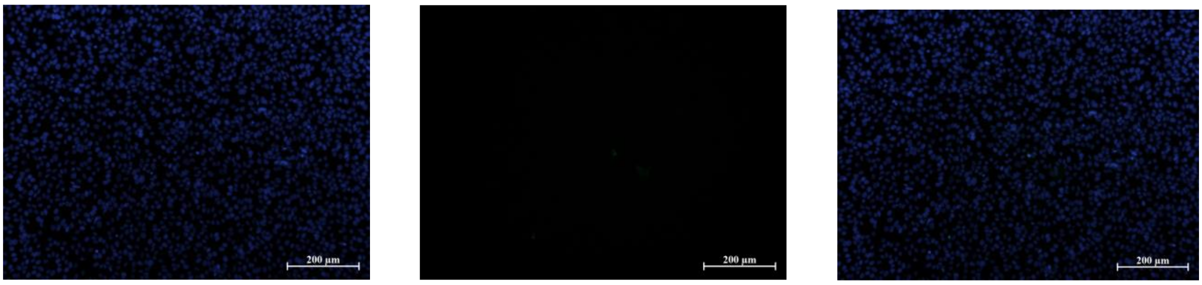


Xanthohumol

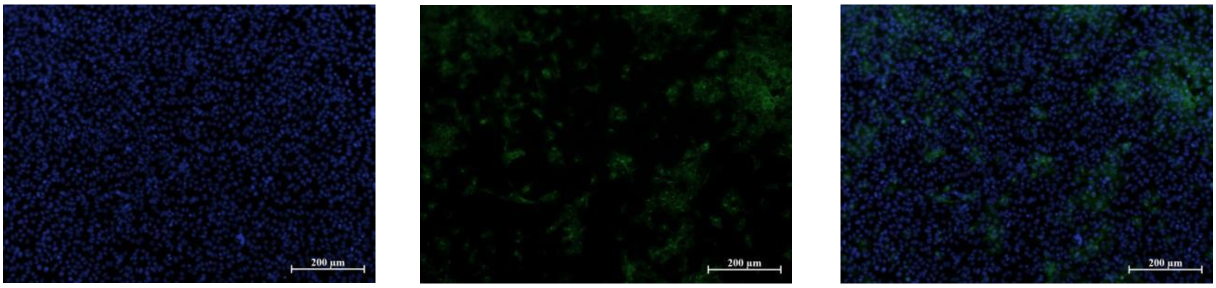


Viral control

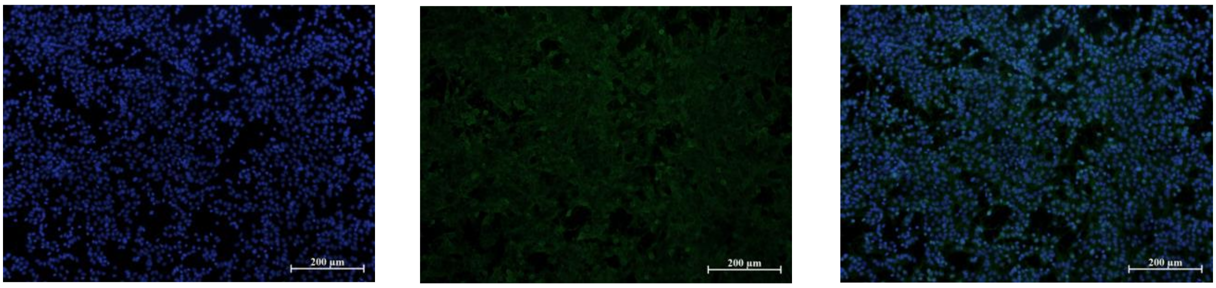


Negative control

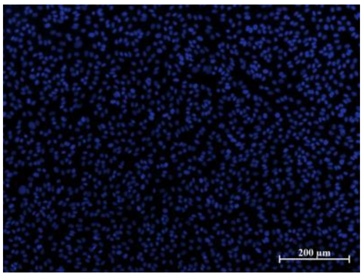
200 µm
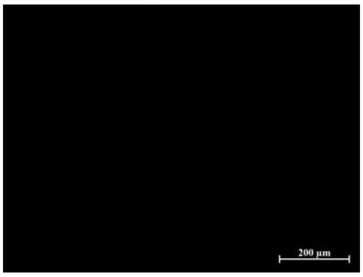
200 µm
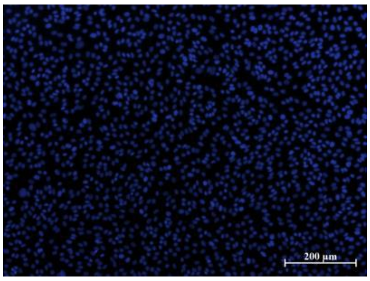
200 µm
